# The impact of mechanical stress on anatomy, morphology, and gene expression in *Urtica dioica* L.

**DOI:** 10.1007/s00425-024-04477-0

**Published:** 2024-07-06

**Authors:** Urszula Zajączkowska, Dominika Dmitruk, Joanna Sekulska-Nalewajko, Jarosław Gocławski, Alicja Dołkin-Lewko, Barbara Łotocka

**Affiliations:** 1 Department of Forest Botany, Warsaw University of Life Sciences, Nowoursynowska 166, 02-776 Warsaw, Poland; 2 Department of Botany, Warsaw University of Life Sciences, Nowoursynowska 166, 02-787 Warsaw, Poland; 3 Institute of Applied Computer Science, Lodz University of Technology, Stefanowskiego 18/22, 90-924 Lodz, Poland

**Keywords:** Environmental stress, Gene expression, Leaves shape analysis, Mechanical impact, Nettle, Thigmomorphogenesis, *TOUCH* genes, *UdTCH1* gene

## Abstract

**Main conclusion:**

Mechanical stress induces distinct anatomical, molecular, and morphological changes in *Urtica dioica*, affecting trichome development, gene expression, and leaf morphology under controlled conditions

**Abstract:**

The experiments were performed on common nettle, a widely known plant characterized by high variability of leaf morphology and responsiveness to mechanical touch. A specially constructed experimental device was used to study the impact of mechanical stress on *Urtica dioica* plants under strictly controlled parameters of the mechanical stimulus (touching) and environment in the growth chamber. The general anatomical structure of the plants that were touched was similar to that of control plants, but the shape of the internodes' cross section was different. Stress-treated plants showed a distinct four-ribbed structure. However, as the internodes progressed, the shape gradually approached a rectangular form. The epidermis of control plants included stinging, glandular and simple setulose trichomes, but plants that were touched had no stinging trichomes, and setulose trichomes accumulated more callose. Cell wall lignification occurred in the older internodes of the control plants compared to stress-treated ones. Gene analysis revealed upregulation of the expression of the *UdTCH1* gene in touched plants compared to control plants. Conversely, the expression of *UdERF4* and *UdTCH4* was downregulated in stressed plants. These data indicate that the nettle's response to mechanical stress reaches the level of regulatory networks of gene expression. Image analysis revealed reduced leaf area, increased asymmetry and altered contours in touched leaves, especially in advanced growth stages, compared to control plants. Our results indicate that mechanical stress triggers various anatomical, molecular, and morphological changes in nettle; however, further interdisciplinary research is needed to better understand the underlying physiological mechanisms.

**Supplementary Information:**

The online version contains supplementary material available at 10.1007/s00425-024-04477-0.

## Introduction

Throughout their lifespan, plants are subjected to permanent mechanical stimulation. The physical force of moving air particles presents a persistent interaction for terrestrial plants (Grace 1988; Gardiner et al. 2016). Additionally, during growth, roots are in constant interaction with soil particles (Martins et al. 2020; Kolb et al. 2022; Mimault et al. 2022). The process of water vapor condensation occurs on the surface of the above-ground organs. Also, precipitation in the form of rain drops, snow or hail particles hits the bodies of entire plants (Yabuki and Migayawa 1970; Gardiner et al. 2016; Manickathan et al. 2022). Apart from the diverse physical forces of an abiotic origin, physical stimuli can also originate from biotic sources, such as the touch of animals or the touch of foreign or own leaves (Farmer et al. 2020).

Thigmomorphogenesis is the term used to describe the growth and differentiation of plants that are directly affected by a sequence of molecular and electrical events initiated by various types of mechanical stimuli (Telewski and Jaffe 1986; Braam 2005; Brenya et al. 2022). The capacity of plants to identify a particular physical stimulus and subsequently trigger (or not) a specific response is a pivotal aspect of their developmental and growth processes (Telewski 2006).

Plant genomes are rich in genes that are involved in stress-response mechanisms. Some of them are specific for one type of stress, while others act more widely. Some of the genes involved in mechanical, touch-response mechanisms are *TOUCH* (*TCH*), *Universal Stress Protein* (*USP*), *Zinc Finger Protein 2* (*ZFP2*), and *Ethylene-Responsive Transcription Factor 4* (*ERF4*). *TOUCH* genes were the first genes discovered to be mechano-controlled. Their expression increases rapidly after mechanical stimulation, with at least a twofold increase (Braam and Davis 1990; Lee et al. 2005). *USP,* first discovered in *Escherichia coli* (VanBogelen et al. 1990), has numerous homologues in other organisms, including plants, which have hundreds of USP domains and sequences resulting in high diversity of USP proteins’ structure. Generally, *USPs* code proteins with ATP-binding USP domains or with USP domains without ATP-binding activity. The proteins may possess a single USP domain, two USP domains in tandem repetition, or the USP domain may be present alongside other functional domains (Kvint et al. 2003). *USPs* are overexpressed in response to different environmental stresses, e.g., mechanical stress, nutrient deficiency, oxidative stress, heat/cold shock, etc. (VanBogelen et al. 1990; Kvint et al. 2003). *ZFP2* was first discovered in *Juglans regia*, and its homologue was also found in *Populus tremula x alba* (Leblanc-Fournier et al. 2008; Martin et al. 2009). It is considered a primary mechanosensitive gene in woody plants. It encodes a Cys2/His2-type two-zinc-fingered transcription factor, whose mRNA accumulation increases rapidly right after a mechanical stimulus (e.g., bending or wounding) and does not increase after other types of abiotic stress factors (e.g., salt, cold; Martin et al. 2009). ERFs are known to bind to a GCC box—an ethylene-responsive element, which is necessary for transcriptional control by ethylene (Ohme-Takagi and Shinshi 1995; Shinshi et al. 1995). Wounding induces rapid increase in the expression of *ERF4* in tobacco (Suzuki et al. 1998; Nishiuchi et al. 2002).

Although the topic of thigmomorphogenesis appears to be gaining increasing interest in scientific research, its understanding is still incomplete because it affects a variety of species that are affected by physical force in a number of ways depending on the materials they are touched with, at unrelated stimulation frequencies and with a variety of forces. Therefore, it is difficult to define the term "mechanical interaction”. Some studies suggest varying physiological and morphological reactions to tactile stimulation. For example, *Plantago major* new leaves change to a narrower shape when growing older if they have been touched by a dust brush (Anten et al. 2010). Further, brushing *Arabidopsis thaliana* increases the flexibility of the inflorescence shoot (Zhdanov et al. 2022). The petiole-transition zone of *Pilea peperomioides* undergoes changes in its mechanical properties when subjected to mechanical stimulation, despite having a similar anatomical structure of the petiole (Langer et al. 2021). Conversely, when subjected to bimodal mechanical stimulation via *Brachypodium distachyon* stick flexure, stem rigidity is enhanced and flowering is postponed (Gladala-Kostarz et al. 2020).

Our research is primarily focused on elucidating the complex phenomenon of thigmomorphogenesis in *Urtica dioica* L. This species represents an excellent system for studying the mechanical forces involved in plant morphogenesis because of its tactile sensitivity and morphological variability. Our aim is to determine the interactive effects of structural and genetic traits when exposed to mechanical stimuli. Particularly, (i) we wanted to see if the mechanical impulse affected the size of the plant; (ii) because under the influence of a mechanical factor the stem was periodically bent we decided to perform anatomical analyses of the stem; (iii) since the leaves were directly affected by the mechanical impulse, we thought it would be useful to see if this affected their morphology; and (iv) eventually, we wanted to investigate which genes were involved in the response to mechanical stress. In our opinion such interdisciplinary strategy aims to decipher the mechanistic basis that shapes plant architecture, thereby improving our understanding of resilience, plasticity and the broader range of adaptive strategies they use.

## Materials and methods

### Experimental set-up

**Fig. 1:**
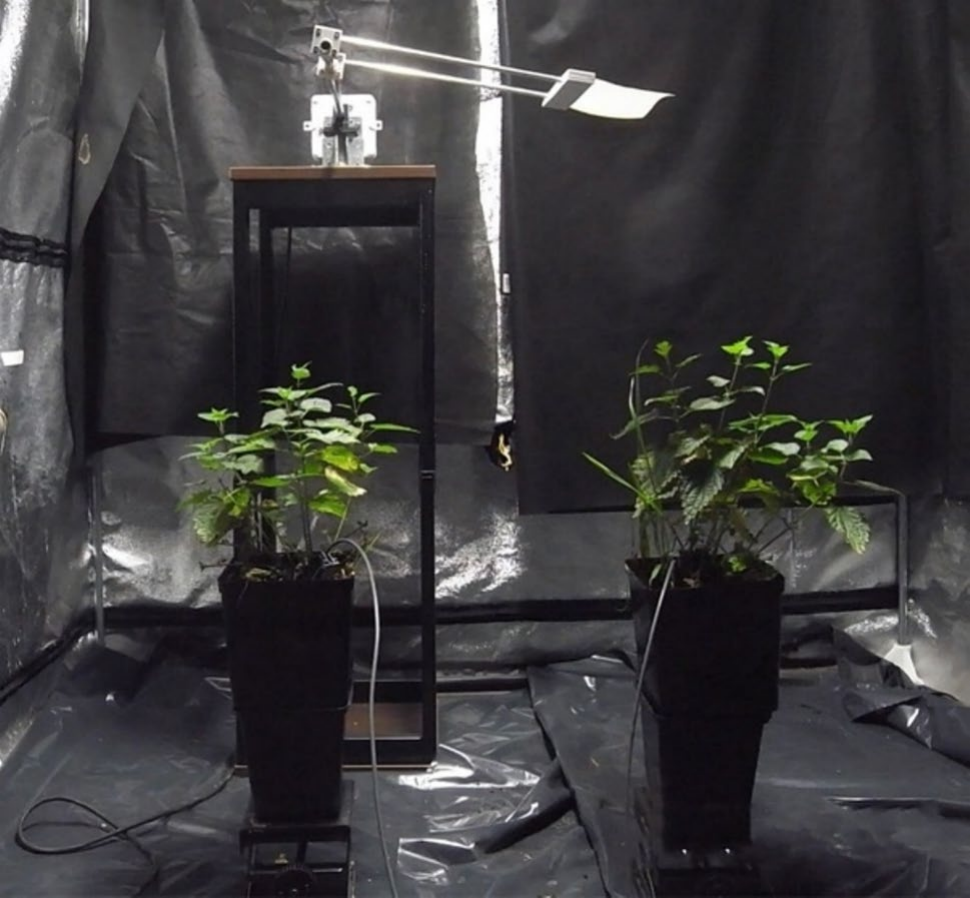
Device designed for mechanical stimulation of nettle plants; rotating arm with paper sheet attached is visible. The plant subjected to stimulation is positioned on the left side, while the control plant is on the right

A single individual of *Urtica dioica* L. was collected in Wołomin (Poland, 52.360773, 21.270523) and was vegetatively propagated by dividing rhizomes into 24 clone plants to exclude genetic variations. The clones were grown outdoors in containers, in a universal substrate (PolLas). Plants were regularly watered. The experiment was carried out at the Department of Forest Botany, at the Warsaw University of Life Sciences, Poland. The clones were placed in a growth-box under constant light (Phytolite HPS 600W lamp, photon flux 1.045 kl mol m^−2^ s^−1^, luminous flux 100 km) and temperature (21 °C) conditions. Mechanical stimulation was affected using a specially designed device with a 40-cm-long rotating arm to which an A5-size, 80 g/m^2^ paper sheet (International Paper POL) was attached. The arm moved continuously at a rotational speed of 3.3 rpm, and the paper gently touched the tops of the plants. Twelve pairs of plants, each consisting of four internodes—touched and control ones—were subjected to the experiment for 10 days (Fig. 1). After the end of the experiment, the height of the plants was measured from frames of recorded video using ImageJ (http://rsbweb.nih.gov/ij/index.html) software (Schneider et al. 2012). The nutation movements of the plants were recorded using a Ricoh GX200 (Japan) camera with a built-in intervalometer. Images were captured using camera lenses oriented perpendicularly to the plant’s axis. The shutter was released every 5 min (Supplementary file S1, time-lapse movie).

### Stem anatomy

For the anatomical studies, four upper internodes (labelled below as: il—the internode below the apical bud; i2, i3, i4—basipetally successive internodes) were taken from both control and stress-treated plants in two biological replicates originating from independent experiments. Whole internodes were fixed in FAA neutralized with CaCO_3_ (Broda 1971) for several days. They were then rinsed with 70% ethanol and stored in its fresh change until further processing. Short fragments of the internodes, after hydration, were cut into sections, 30–70 µm thick, using a vibratome VT l000S (Leica); also, some hand sections were used, if needed. First, unstained sections were examined as controls for histochemical tests. Next, the same sections were dark-incubated for at least 5–10 min in a solution of 0.1% (w/v) aniline blue (Chempur, Piekary Śląskie, Poland) in 0.15 M K_2_HPO_4_ (Sigma), mounted in a drop of the same solution and viewed using UV excitation for the detection of callose (and autofluorescence). Another section of the same sample was treated with commercial Herzberg’s reagent (Pol­Aura Sp. z o.o., Morąg, Poland) to distinguish the lignified cell. Sections of i4 were stained with Astra blue–fuchsin–chrysoidin (Etzold 2002) to confirm general anatomical conclusions.

The observations were carried out using a Provis AX70 light microscope, in a bright field (BF) or epifluorescence configuration, equipped with a dedicated digital camera UC90 (both from the Olympus Corporation) controlled by cellSens Standard 1.18 (SIS) software. For (auto)fluorescence observations, the Narrow Ultraviolet (NU) cube was used (excitation filter 360–370 nm + dichroic mirror 420 nm + barrier filter > 420 nm). Images were saved as LZW-compressed tiff files of 3384 × 2708 pixel resolution and processed using Adobe Photoshop CS7 Extended (Adobe Systems Inc.) software. Images of uneven sections were prepared by means of focus-stitching several optical “sections” (Auto-Blend Layers tool), and images of large areas (e.g., whole sections) were obtained by combining several images using the Automatize-Photomerge or Panorama tools. Then, the Levels and Curves tools were used to improve image clarity. For publication purposes, microscopic documentation was edited in CorelDRAw®2020 (Corel Corp.) software.

### Identification of *U. dioica* stress-related proteins

Orthologs of *A. thaliana* mechanical stress-related proteins were identified in the *U. dioica* genome. Utilizing NCBI (NCBI Resource Coordinators 2018) and UniProt (The UniProt Consortium 2017) databases, sequences from *A. thaliana* were aligned against genomes of *Morus notabilis*, *Prunus* sp. or *Vitis vinifera* using BLASTp, serving as a comparative basis for phylogenetic analyses conducted on the Phylogeny.fr platform and MEGA X. Despite no novel insights from the phylogenetic trees for most proteins, closer orthologues for ERF4 and TCH3 were determined in *M. notabilis*, guiding further examination of *U. dioica* orthologues. Sequences of mechanical stress-related proteins of *A. thaliana* and corresponding sequences of *U. dioica* chosen for further analyses are presented in Table 1.

**Table 1:** Sequences of mechanical stress-related proteins of *A. thaliana* and corresponding sequences of *U. dioica* chosen for further analyses

*Arabidopsis thaliana*		*Urtica dioica*	
Acronym	Identification number	Protein name in China National GeneBank database	Gene name in this study
ERF4	NP_188139.1	gnl|onekp|WKCY_scaffold_2048012	*UdERF4*
USP	Q8LGG8	gnl|onekp|WKCY_scaffold_2008388	*UdUSP*
ZFP2	NP_200560.1	gnl|onekp|WKCY_scaffold_2005901	*UdZFP2*
TCH1	NP_850344.1	gnl|onekp|WKCY_scaffold_2006738	*UdTCH1*
TCH3	NP_001189723.1		
TCH2	NP_001318691.1	gnl|onekp|WKCY_scaffold_2046871	*UdTCH2*
TCH4	NP_200564.1	gnl|onekp|WKCY_scaffold_2003976	*UdTCH4*

More descriptions of this part are available in the Supplementary file S2 (including Figs. S1–S3 and Tables S1–S3).

### RNA isolation and cDNA synthesis

The RNA was isolated from *U. dioica* leaves in six biological replicates. Total RNA isolation was performed using the TRIZOL method (Chomczynski and Sacchi 1987) with additional DNase digestion (TURBO DNA-*free*™ kit, Invitrogen™). RNA concentration and purity were tested by the spectrophotometric method with BioSpectrometer kinetic (Eppendorf) with a µCuvette adapter (Eppendorf), while its integrity was tested by electrophoretic separation in 1% agarose gel. An equivalent amount of each RNA sample was used for cDNA synthesis with a High-Capacity cDNA Reverse Transcription Kit (Thermo Fisher Scientific, Waltham, MA, USA).

### Reverse transcriptase PCR (RT-PCR) and real-time qPCR

The protein sequences obtained using BLASTp were translated in silico to the most probable DNA sequences with Sequence Manipulation Suite (Stothard 2000). Primers were designed with the Primer3 software (Primer3Plus, Free Software Foundation, Inc., Boston, MA, USA) (Untergasser et al. 2007). Primer sequences are presented in Table 2. Each primer pair was tested with reverse transcriptase PCR (RT-PCR) (Fig. S3b, c), using reaction conditions as presented in Table S3. The *UdTHC2* and *UdUSP* primers did not result in any products. Two sets of new *USP* primers were designed: *UdUSP-a* and *UdUSP-b*, they were tested as specific (Fig. S3d) and successively used for qPCR.

**Table 2:** Sequences of primers designed for RT-PCR and real-time qPCR

*Urtica dioica* gene	Primer	Sequence (5′ → 3′)	Product length (bp)
*UdERF4*	Forward	CTGGGCACCTTTGATACCC	90
	Reverse	AAAGTTGGTTTTCGCTTTCG	
*UdUSP*	Forward	ATGTGAACAACGAAACCGGC	107
	Reverse	AATGCCGCCTTTGGTTTTCG	
*UdZFP2*	Forward	CGCGCGTGTTTAGCTGCAA	103
	Reverse	CGTTTCGCCATGGTGCGTTC	
*UdTCH1*	Forward	GCGAAAAACTGACCGATGAT	106
	Reverse	TTCGCCATCATCACTTTCAC	
*UdTCH2*	Forward	GGCAACGATACCAACAAAGAA	101
	Reverse	TTTTTCAGCACGCTATGCAG	
*UdTCH4*	Forward	CGTGACCGCGTATTATCTGA	108
	Reverse	AACACGTTGGTATGCAGCAC	
*Ud-cyclophilin*	Forward	TTGGAGCCCTGAGACAATTC	99
	Reverse	ACGGAGGTTTTGCACTTCAC	
*Ud-tubulin*	Forward	GGGCTTCTTGGTCTTCAATG	80
	Reverse	TCGACAGACAAACGTTCGAG	
*Ud-actin*	Forward	TCCGATTTACGAGGGTTACG	98
	Reverse	GCTCCGTCAGGATTTTCATC	
*UdEF2*	Forward	AGAGTTGCGTCGGATTATGG	128
	Reverse	CTTCTTGGGCAATGATACCC	
*Ud-eTIF4E*	Forward	AGGTGAGCATTGGGAAACAG	103
	Reverse	TTCTTGGCACCCCTATCAAG	
*Ud-histone3*	Forward	AGGAAGATGGCAAGCTGAAG	3993
	Reverse	ATTGCACACAGCATCGAGTC	
*UdTIP41*	Forward	TCCCCAACCCTTAAACTGTG	71
	Reverse	CTTTCCCCAAACACCATCTC	
*UdRAN*	Forward	AAGAGCGGACGAAGAATACG	93
	Reverse	CGAGTGAATCGGGAAAGAAG	
*UdCDPK*	Forward	GATGTGGATGGGAATGGAAC	143
	Reverse	TCGTCTCGGGTGATAAATCC	
*UdUSP-a*	Forward	GCCTGCTGTTTAACGCGATT	104
	Reverse	GCCAATCGCTTCTTTCGCTT	
*UdUSP-b*	Forward	ATGTGAACAACGAAACCGGC	107
	Reverse	AATGCCGCCTTTGGTTTTCG	

Real-time qPCR was conducted in the CFX Connect Real-Time PCR Detection System (Bio-Rad, Hercules, CA, USA) using iTaq™ Universal SYBR® Green Supermic (Bio-Rad, Hercules) according to the manufacturer’s instructions. Each reaction was performed in six biological replicates and two technical repetitions. Reaction conditions were as follows: 2 min at 95 °C, 40 cycles of 15 s at 95 °C/30 s at 60 °C. For the melting curve, these were: 60 °C gradually increasing to 95 °C in 0.5 °C increments, 5 s per step. The specificity of each primer pair was verified using melting curve analysis.

Real-time qPCR efficiency was calculated with the help of the LinRegPCR tool (Ramakers et al. 2003). The relative transcript accumulation level was calculated using the single-delta method (Livak and Schmittgen 2001). The statistical significance of differences in transcript accumulation levels was calculated using Student’s t-test. As potential reference genes, the following were tested with RT-PCR: nine published genes coding for cyclophilin, tubulin, actin, the translation elongation factor EF2, the eukaryotic translation initiation factor eTIF4E, histone3, the tonoplast intrinsic protein TIP41, the gene coding for small GTP-binding protein RAN and the gene corresponding to the calcium-dependent protein kinase CDPK (Backes et al. 2018). The four most stable candidates: tubulin-, RAN-, actin- and TIP41-encoding genes, were tested with qPCR. For normalization of the qPCR results, tubulin- and actin-encoding genes were chosen as the reference.

### Shape analysis of nettle leaves under the influence of mechanical stress

**Fig. 2:**
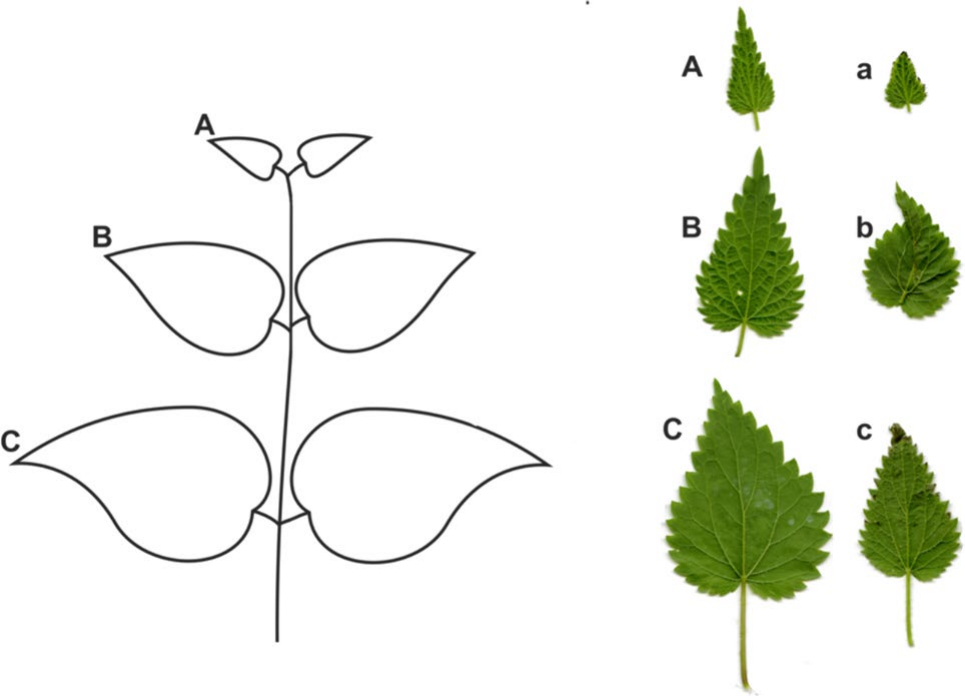
Scheme of foliage markings and sample images of nettle leaves growing at the top nodes: A–C, untouched control leaves, a–c, touched leaves after a mechanical stimulus

The study of nettle leaves was carried out on seven replicate plant specimens both for untouched leaves and leaves under the influence of a mechanical stimulus. For the leaves growing at the top, six separate node images were captured. As shown in Fig. 2, these leaves are marked from the top of the plant with the letters: a–c or A–C. A lower case letter (e.g., a) denotes leaves after the action of a mechanical stimulus, and a capital letter denotes untouched leaves (from corresponding nodes of control plants). The leaf blades from the same plant, spread out on the scanner glass against a uniform white background, were captured with a resolution of 1200 dpi and saved into JPEG files. Next, the images including single leaves were carefully cropped from the original scans using Adobe® Photoshop® 6.0 and saved in separate files.

A total of 84 images of individual leaves were analyzed. Most of the analysis was done by comparing the leaves in pairs: touched leaf–untouched leaf. These leaves had to be located at an analogous distance from the top of the plant (for example, a with A). At the same time, control leaves from the same nodes were also compared in pairs to capture natural variations in leaf morphology. The leaf petiole, which should not be subject to surface and shape analysis, was removed from the leaf image with the help of an interactive method built into the measurement algorithm.

**Fig. 3:**
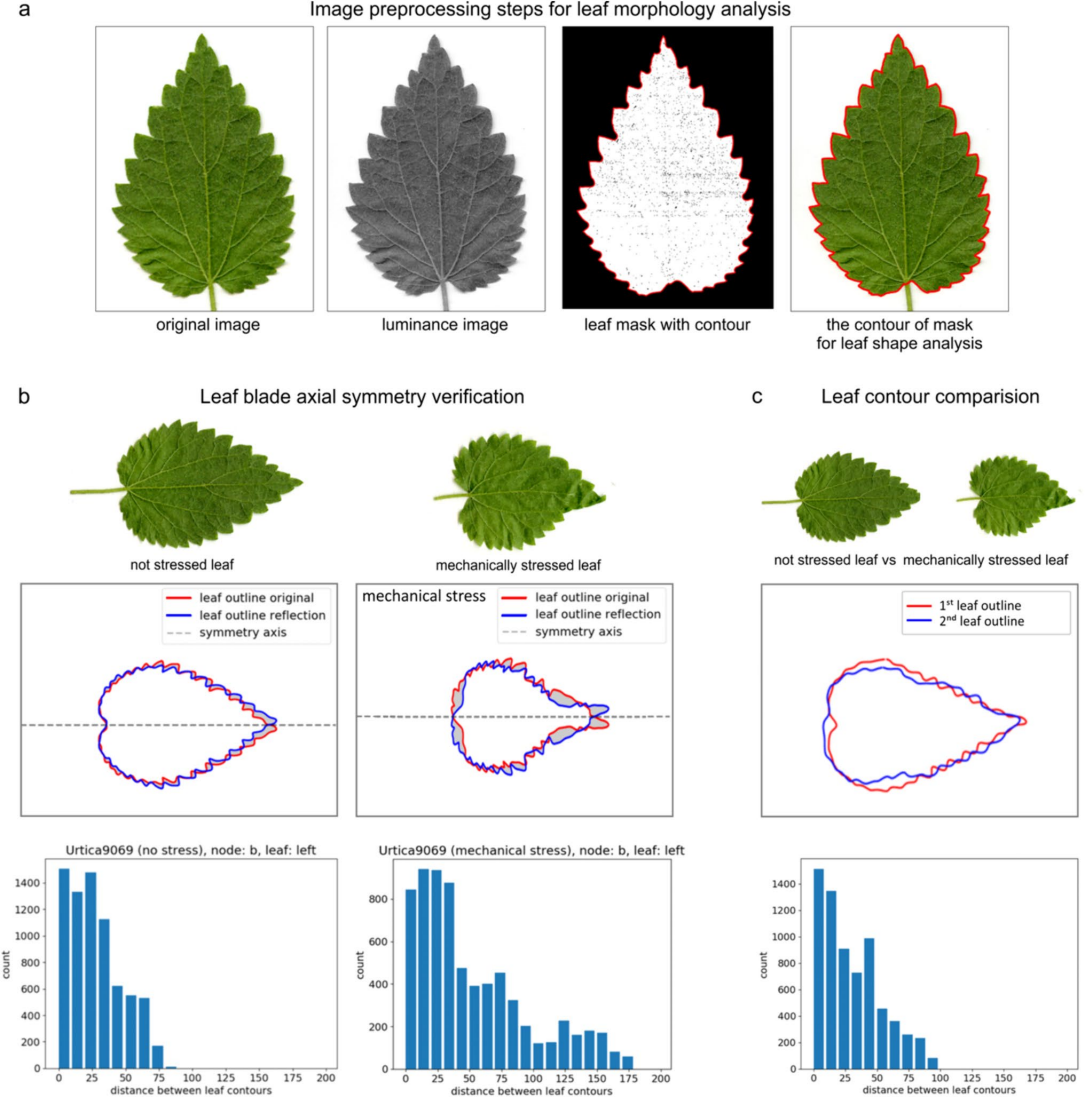
Leaf image processing and shape analysis in not stressed (control) and mechanically stressed (touched) leaves. **a** The main steps of the algorithm, aimed at obtaining a mask from the image of a leaf and determining its outline. **b** Illustration of the leaf blade axial symmetry verification. The original leaf blade contour (red) is compared with its mapping in axial symmetry (blue). **c** Illustration of comparing the contours of two leaves after positioning, size normalization and denoising using the EFD, EFD-1 transform sequence. Scaling of the large diameter of the 1st harmonic ellipse of each contour to the dimension of 1000 image points was applied. The histograms represent the distance of the compared contours. The horizontal scale of the histogram represents the distance between contours (in image points), and the vertical scale represents the number of points at a given distance

In order to assess the changes in leaf morphology, an image analysis algorithm has been developed in the Python language environment, which aims at the area and shape comparison of leaves. The main steps of the algorithm are illustrated in Fig. 3. The pre-processing stage of the algorithm loads an RGB leaf image from its JPEG file into the program workspace, then transforms it into a YUV luminance image, which in turn is converted to a binary leaf mask using the Otsu thresholding method (Otsu 1979) (Fig. 3a). This mask image is then cleared of black holes inside the leaf and small, white external areas, using image morphology operations (Serra and Soille 1994; Gonzales and Woods 2018). The corrected mask is outlined, using functions from the *OpenCV* library (OpenCV Open Source Computer Vision, https://docs.opencv.org/4.x), and its contour plays a key role in leaf shape analysis.

Qualitative and quantitative indicators used for determining leaf morphology, obtained with image processing methods, were as follows:

- leaf surface area and the relative percentage of change in the leaf area versus the untouched leaf from the same plant;
- measures of shape difference derived from a pairwise comparison of denoised and normalized leaf contours obtained by EFD (Elliptic Fourier Descriptors) (Kuhl and Giardinam 1982);
- measures of deviation from the axial symmetry of leaf contours denoised and normalized by EFD.

### Leaf surface area analysis

After calculating the leaf area, expressed in cm^2^, the relative difference of the leaf area in pairs was examined. The leaves were examined in pairs: one type of pair was the untouched leaves from the same node, paired randomly, and the second type of pair was composed of an untouched and a touched leaf from corresponding node.

The mean relative difference ${\overline{\delta }}_{a}$ in the leaf area was calculated according to the equation below.

1 $${\delta }_{a}\left(i\right)=2*\frac{\left|area\left(i\right)-are{a}^{\prime}\left(i\right)\right|}{area\left(i\right)+are{a}^{\prime}\left(i\right)}, \quad { \overline{\delta }}_{a}=\frac{1}{N}\sum\limits_{i=1}^{N}{\delta }_{a}\left(i\right),$$

where $area\left(i\right)$ and $area^{\prime}\left(i\right)$ denote compared leaf areas, and *N* represents the number of pairs of leaves to be compared.

### Leaf shape analysis

To assess the shape, a Fourier transform of the leaf blade contours was performed with the EFD method (Kuhl and Giardina 1982), which was designed to track and analyz the contours of plant organs (Kincaid and Schneider 1983; Iwata et al. 1998, 2002). Carrying out a simple and inverse EFD transformation of the leaf contours together with normalization in the frequency domain allowed the unification of the size and position of all examined objects, a condition for comparing only their shapes. To obtain the correct mapping of the contours with reduced noise, the initial 50 harmonics were used. An example of matching the contours of two leaves after normalization is shown in Fig. 3c.

On the plane of normalized leaves, the average distance ${d}_{avg}$ and maximum distance ${d}_{max}$ between their contours, ${C}_{1}$ and ${C}_{2}$, were computed for each pair. First, the values of contour shape difference indicators ${d}_{avg}$ and ${d}_{max}$ were averaged for the pairs in individual categories of nodes: untouched leaf—touched leaf (a–A, b–B, c–C) and control leaf—control leaf (A–A, B–B, C–C).

The distance between the leaf contours was also described in the EFD transform space as ${D}_{\text{1,2}}$—the sum of the squared differences in Fourier coefficients of the shapes ${C}_{1}$ and ${C}_{2},$ in the range from the first to the *K*-th harmonic of each contour.

2 $${D}^{2}= {D}_{\text{1,2}}^{2} =\frac{1}{K}\sum_{i=1}^{K}{\left({a}_{1,i}-{a}_{2,i}\right)}^{2}+{\left({b}_{1,i}-{b}_{2,i}\right)}^{2}+{\left({c}_{1,i}-{c}_{2,i}\right)}^{2}+{\left({d}_{1,i}-{d}_{2,i}\right)}^{2},$$

where ${a}_{n,i}, { b}_{n,i ,} {c}_{n,i}, { d}_{n,i}$ are the Fourier coefficients of the shapes ${C}_{n}$, *n* = {1,2} calculated by the transform for the *i*-th harmonic.

### Leaf symmetry analysis

The symmetry of nettle leaves can be disturbed under the influence of touch during growth. In order to define symmetry errors, the same approach as for shape analysis was used. EFD transform with the first harmonic phase normalization allowed to establish the symmetry axis in the leaf outline image as a horizontal line (Fig. 3b) determined by the major axis of the basic ellipse. To calculate the symmetry error, two contours were compared: the leaf contour and its appearance obtained after the mirror reflection (blue line). The first of the proposed leaf blade symmetry errors—error ${e}_{d}$—was defined in Eq. (3) as the mean distance between the leaf original outline $C$ and its axial symmetry mapping ${C}^{\prime},$ both obtained after EFD forward and backward transform with normalization.

3 $${e}_{d}=\frac{1}{{n}_{l}}\sum\limits_{k=1}^{n}{\Vert {C}_{k}^{\prime}-C\Vert }_{2},$$

where $n$ is the number of contour points, and ${\Vert \cdot\Vert }_{2}$ denotes the Euclidean distance in the leaf image plane. Selected histograms of outline distances for the leaves are shown in Figs. 3c and 5b. The second suggested symmetry error ${\varepsilon }_{a}$ was expressed in Eq. (4) as the ratio of area difference between the leaf blade and its symmetrical mapping referred to the leaf area.

4 $${\varepsilon }_{a}=\frac{area\left({C}^{\prime}-C\right)}{area(C)},$$

where $area(C)$ denotes the area inside contour $C$, and $area\left({C}^{\prime}-C\right)$ is the area enclosed between the contours $C$ and $C^{\prime}$ (Fig. 3b).

### Statistical analyses

The obtained data were statistically analyzed using the following tests: the two-tailed Student's *t*-test was used to test the hypothesis equality of the means of two samples assuming unequal variances; the Shapiro–Wilk test was applied to test the normality of the distribution; and the Mann–Whitney test was used to verify that two populations not normally distributed are significantly different at the significance level of 0.05.

## Results

### Stem height

**Fig. 4:**
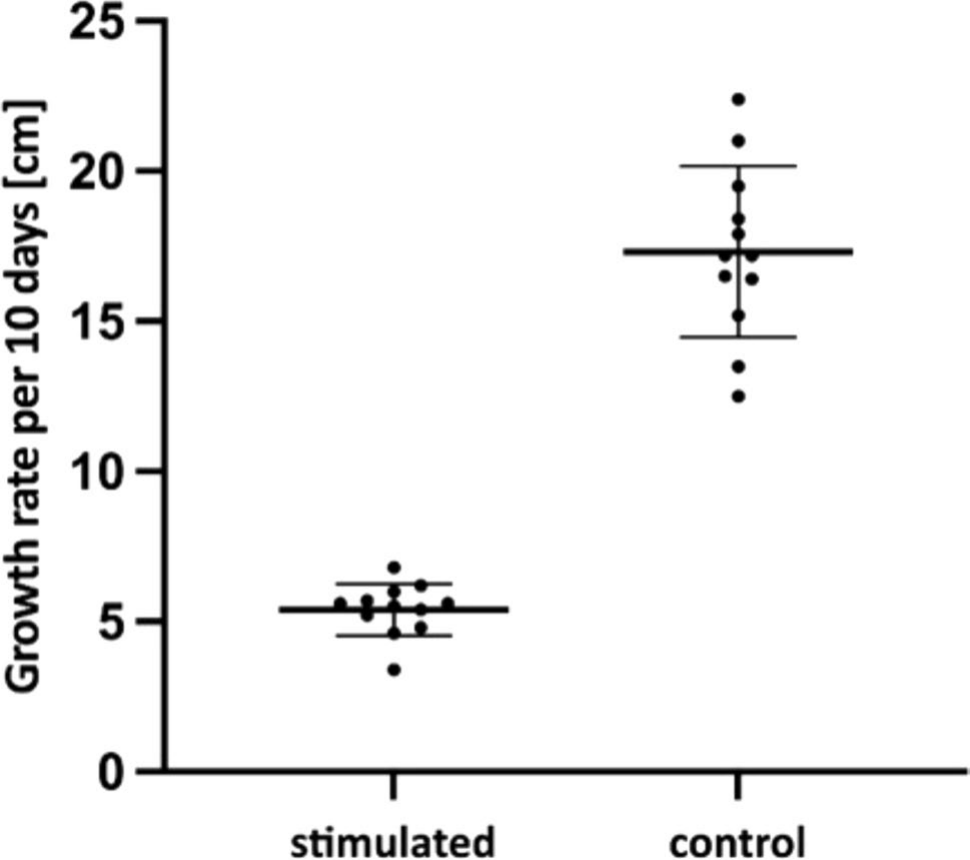
Height of *Urtica dioica* plants subjected to mechanical stimuli (touching) in comparison with the control plants. The average height of the mechanically stressed plants was 5.40 cm (SD ± 0.86, *n* = 12) and the control was 17.31 cm (SD ± 2.85*, n* = 12)

Mechanical stress had a limiting effect on *U. dioica* plant height growth. The height differences between control and stimulated groups, was tested using an unpaired t-test. We found a significant height difference (*P* < 0.0001, *t* = 13.85, df = 22), with the stimulated group averaging 5.4 cm and the control group 17.31 cm. The effect size was substantial (*R* squared = 0.897), indicating a pronounced impact of stimulation. Variance comparison further supported these findings (*F* = 10.94, *P* = 0.0004). This evidence strongly suggests the stimulus's significant influence on height disparities between the two groups (Fig. 4; Supplementary file S2, Table S4).

### Stem anatomy

**Fig. 5:**
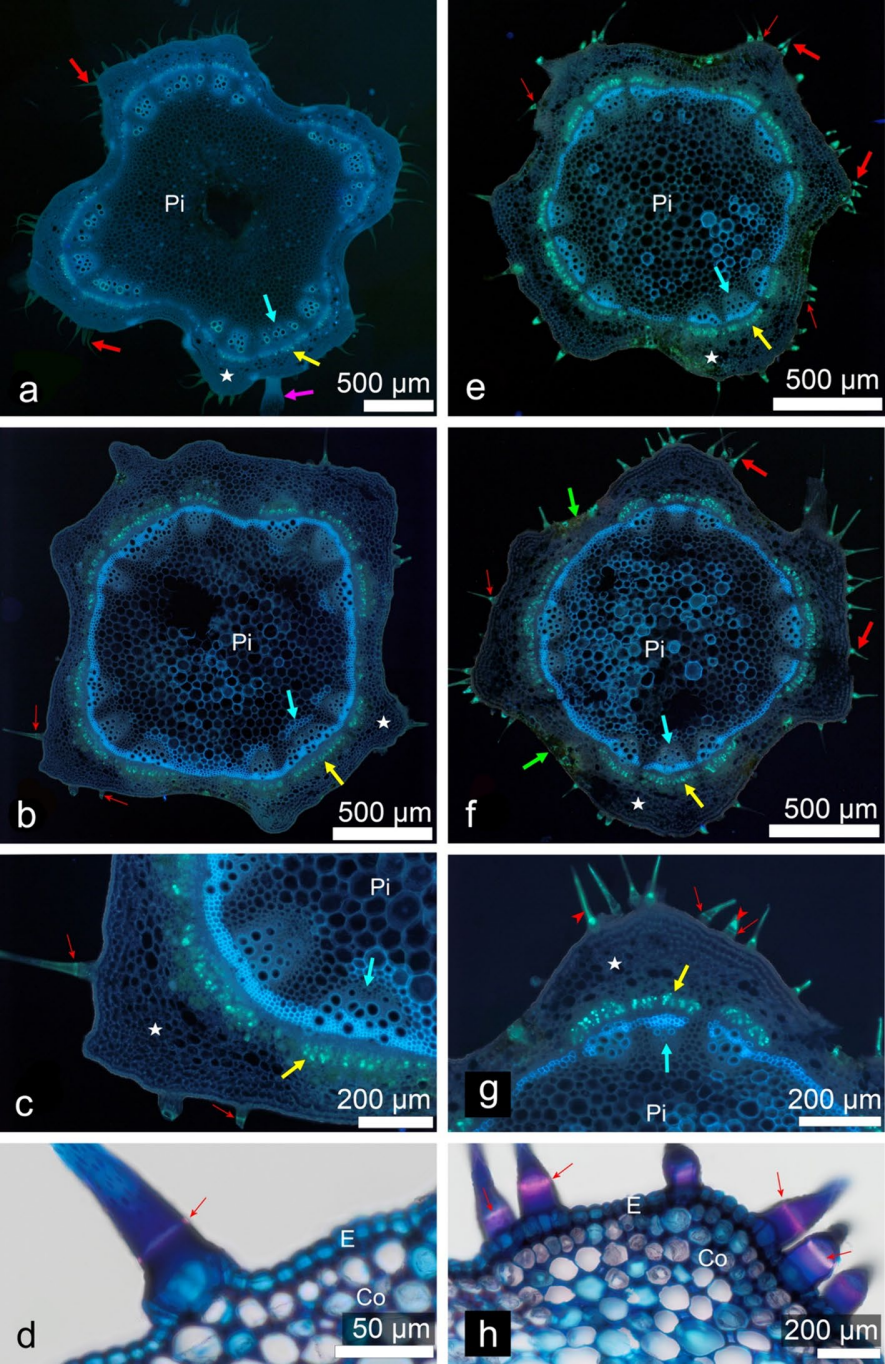
Changes in the anatomical structure of the internodes in control (**a**–**d**) and mechanically stressed (**e**–**h**) *Urtica dioica* plants; cross sections of internodes No. 3 (**a**, **e**) and No. 4 (**b**–**d**, **f**–**h**). UV-excited autofluorescence combined with callose-associated aniline blue fluorescence (**a**–**c**, **e**–**g**) or Etzold's staining (**d**, **h**). Co, collenchyma; E, epidermis; Pi, pith parenchyma (note the difference between control and stressed plants in appearance of parenchyma cells with lignified cell walls discernible due to their strong blue autofluorescence); red thick arrows, setulose trichomes; red slim arrows, Ortmannová’s ring in setulose trichomes; red arrowheads, large deposits of callose in trichomes visualized using aniline blue fluorescence; pink arrow, base of the stinging trichome; asterisk, primary cortex; yellow arrows, phloem location indicated with fluorescence of callose associated with sieve elements; blue arrows, xylem (arrows point the position of the primary xylem in the original vascular bundles of the stem primary structure); green arrows, sectors of mechanically damaged epidermis and cortex

**Fig. 6:**
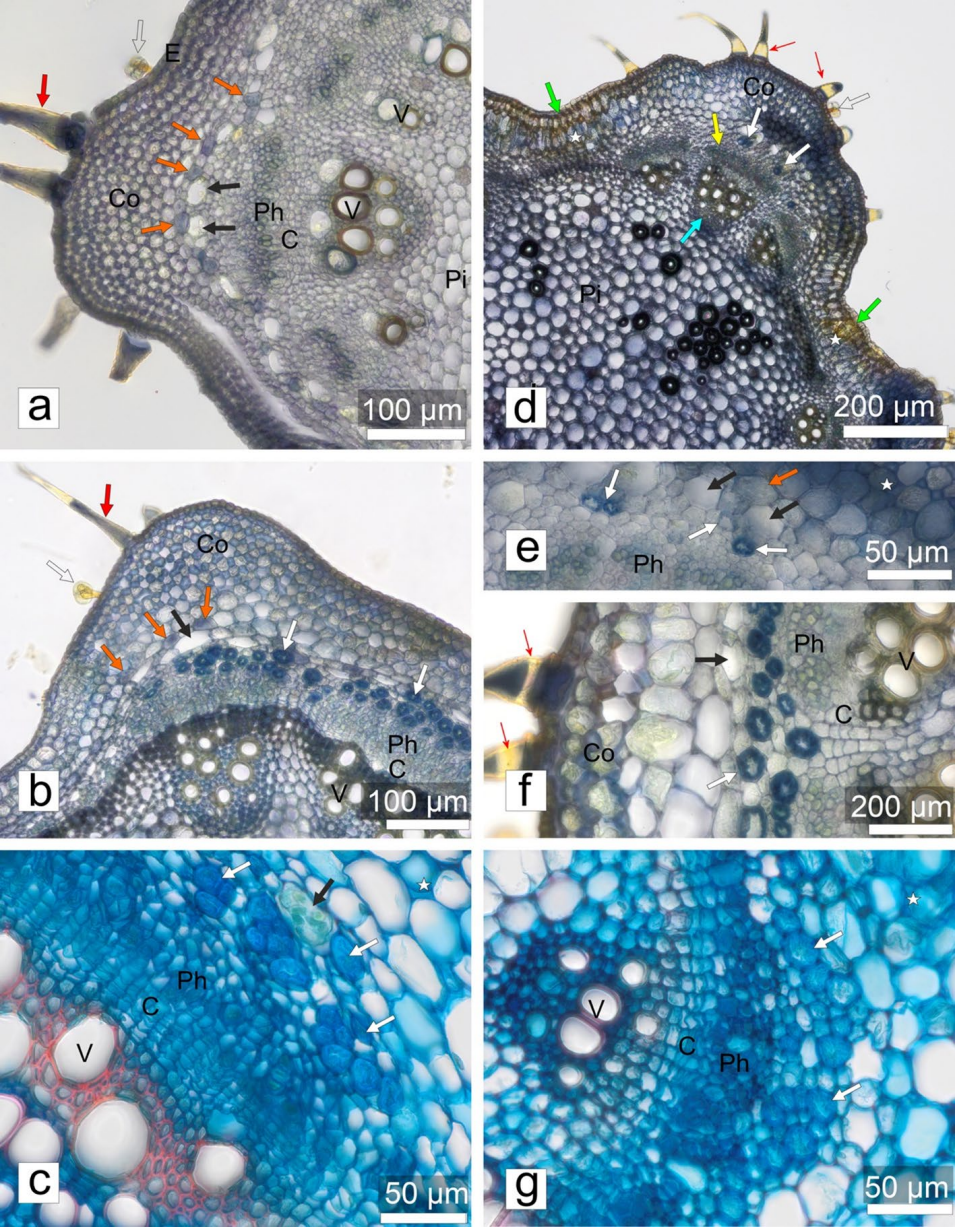
Details of internode structure in control (**a**–**c**) and mechanically stressed (**d**–**g**) *Urtica dioica* plants. Cross-sections of internodes No. 2 (**a**, **d**, **e**), No. 3 (**b**, **f**) and No. 4 (**c**, **g**). Sections stained by Herzberg reagent (**a**–**b**, **d**–**f**) or Etzold's method (**c**, **g**). C, cambium; Co, collenchyma; E, epidermis; Ph, phloem; Pi, pith parenchyma; V, vessels; empty arrows, glandular trichomes; red thick arrows, setulose trichomes; red slim arrows, Ortmannová’s ring in setulose trichomes; asterisk, primary cortex; yellow arrow, phloem; blue arrow, xylem; orange arrows, starch sheath cells with statolith starch stained violet; black arrows, laticifers (note presence of latex in **c**); white arrows, pericyclic fibers; green arrows, sectors of mechanically damaged epidermis and cortex

The general anatomical structure of the stems of control and stress-treated plants was similar. The shape of the internode cross sections was distinctly 4-ribbed in i1 (Figs. 1e, 5a), with flat ribs alternating with convex ones, gradually approaching a rectangular shape in i4 (Figs. 1f, 5b). This was mainly due to the secondary growth of vascular tissues. The uniseriate epidermis covered the internodes. No stomata were found in any section examined. The epidermis in control plants included trichomes of three types: stinging, glandular and simple setulose ones, the latter in two sizes. Trichome density was high in i1 and gradually diminished in i2–i4, in accordance with the circumferential growth of the stem. All types of trichomes were already present in i1. The stinging trichomes were the least frequent, and they consisted of a massive, multicellular base with a strongly birefringent cell walls. In stress-treated plants, the stinging trichomes were not found in any section of all internodes examined. The setulose trichomes accumulated more callose than control plants (Fig. 5c, g). As early as in i1, bright, spot signals of aniline blue fluorescence were visible in numerous setulose trichomes, and they were all weakly but uniformly fluorescent (Fig. 5e). As revealed by Herzberg's staining in control plants, setulose trichome cell walls were initially non-lignified (stained blue) and covered by cuticle stained brightly yellow that separated from the cell wall at the trichome bases after prolonged incubation in Herzberg's reagent (Fig. 6a). In setulose trichomes from older internodes (some i3, most i4), the cell wall had usually a pale yellowish color after Herzberg's staining, which was interpreted as the onset of its lignification (Fig. 6b). In most setulose trichomes (i2–i4), callose deposition detected with aniline blue fluorescence was gradually restricted to the Ortmannová’s ring (Kulich et al. 2015) (Fig. 5b–c, e–g). The ring was also observed after Etzold’s staining as a pink ring in the otherwise blue cell wall (Fig. 5d, h). In stress-treated plants, in all of the older internodes starting with i2, setulose trichomes contained not only a fully formed Ortmannová’s ring but also heavy deposits of callose, located tip wards to the ring (Fig. 5e–g), often obscuring it.

The thickening of the pericyclic fiber cell wall was first recognizable in some i3 sections, while it was evident in all i4 ones. In the fiber of stressed plants, differentiation of the secondary cell wall could occur as early as in i2 in one or two ribs or in a whole section, while in the respective i3 rib, it reached a stage similar to i4 in control plants. The fiber cell wall remained non-lignified in i4, as in control plants (Fig. 6b–g).

In all samples from stress-treated plants, mechanically damaged spots encompassing the epidermis and cortical tissues occurred in i2–i3. In such damaged places, cell walls were thickened and yellowish after Herzberg's staining, indicating lignification (Fig. 6d).

### Gene expression analysis with real-time qPCR

To investigate whether any of the six studied *U. dioica* genes plays a role in plant responses to mechanical stress, the analysis of their expression in control and stressed plant leaves was performed. Despite working with RT-PCR, primers for *UdUSP-a* and *UdUSP-b* did not give any product in qPCR or the C_q_ value was higher than 35 (while negative controls were fine and the reference genes’ C_q_ value was ca. 23), suggesting marginal or no expression of the selected *USP* gene.

**Fig. 7:**
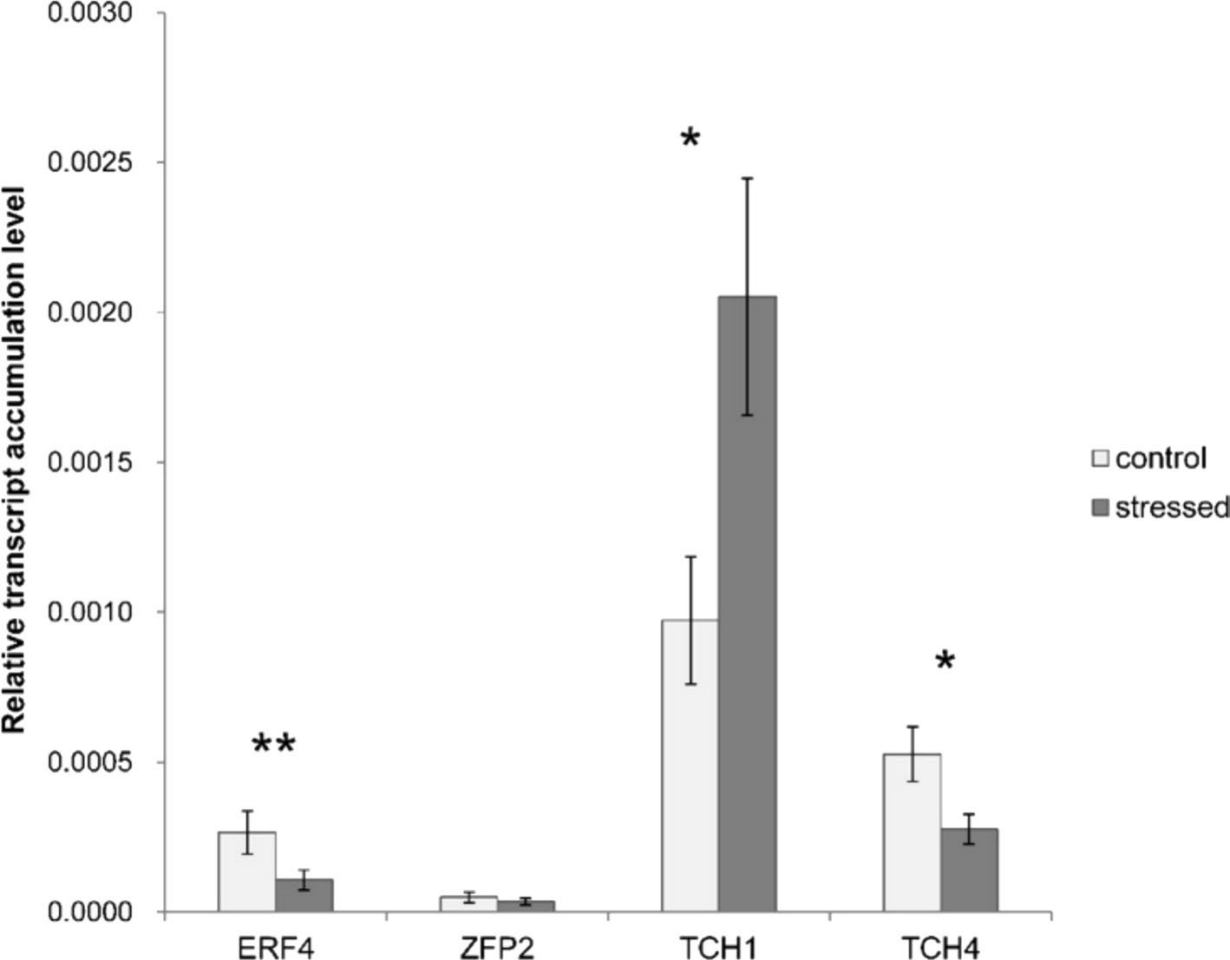
Normalized expression level of *U. dioica ERF4, ZFP2, TCH1* and *TCH4* in control and stressed plants. Mean values (± standard error, SE) are derived from six biological replicates, for which two individual quantitative polymerase chain reactions (qPCR) were performed (*n* = 12). Asterisks above the bars represent statistically significant difference at the level *P* < 0.05 (*) or *P* < 0.01 (**)

The highest-expressed gene was *UdTCH1*, with an expression level ca. 3–5 times higher than that of other studied genes (Fig. 7). Its expression was upregulated twofold in stressed plants compared to control plants, and this difference was statistically significant.

The expression of other genes was much lower, and *UdERF4* and *UdTCH4* were downregulated in stressed plants with statistically significant differences. Their expression in stressed plants constituted half of that in control plants. The expression of *UdZFP2* did not differ between stressed plants and control ones and was close to zero.

### Leaf morphology evaluation

Qualitative and quantitative indicators used for determining leaf morphology are presented in Table 3. These are: (i) leaf area of the control and stressed leaf groups; (ii) leaf symmetry errors expressed by the mean distance or area enclosed between the original contour and its axial symmetry image; (iii) leaf shape variations in pairs expressed by the distance or area enclosed between the contours in the image space; and (iv) leaf shape variations in pairs defined as the square distance of the leaf contours in the EFD transform space.

**Table 3:** Nettle leaves characteristics obtained with image processing methods

	Stressed leaves			Control leaves		
	Leaf nodes					
	a	b	c	A	B	C
*n*	14	14	14	14	14	14
Leaf morphology						
Mean leaf area [cm^2^]	4.03 ± 3.16	6.66 ± 5.25	10.31 ± 4.54	4.67 ± 3.09	22.61 ± 7.40	23.96 ± 12.00
Leaf symmetry indices						
Symmetry error ${e}_{d}$ − contour distance*	84.13 ± 27.65	89.76 ± 30.16	67.78 ± 27.44	47.95 ± 16.40	52.34 ± 32.15	52.51 ± 23.11
Symmetry error ${\varepsilon }_{a}$ − area difference*	58.36 ± 19.98	62.30 ± 23.19	47.51 ± 20.19	33.03 ± 13.49	35.89 ± 21.68	37.63 ± 16.85

	Stressed vs. control leaves			Control vs. control leaves		
	a–A	b–B	c–C	A–A	B–B	C–C
*n* (pairs)	14	14	14	7	7	7
Pairwise comparisons						
Relative area difference *d*_*a*_ [%]	82.61	77.03	55.18	16.21	15.36	11.37
Maximal contour distance *d*_max_*	334.05	290.89	317.90	177.13	179.21	174.86
Average contour distance *d*_avg_*	124.97	97.51	108.15	51.00	56.91	53.88
Squared distance in the space of the EFD transform *D*^*2*^	0.0994	0.0635	0.0750	0.0193	0.0289	0.0274

The symbol ± refers to the standard deviations of preceding mean values, *n* is the number of observations; *, the unit is standardized image point

### Leaf surface

**Fig. 8:**
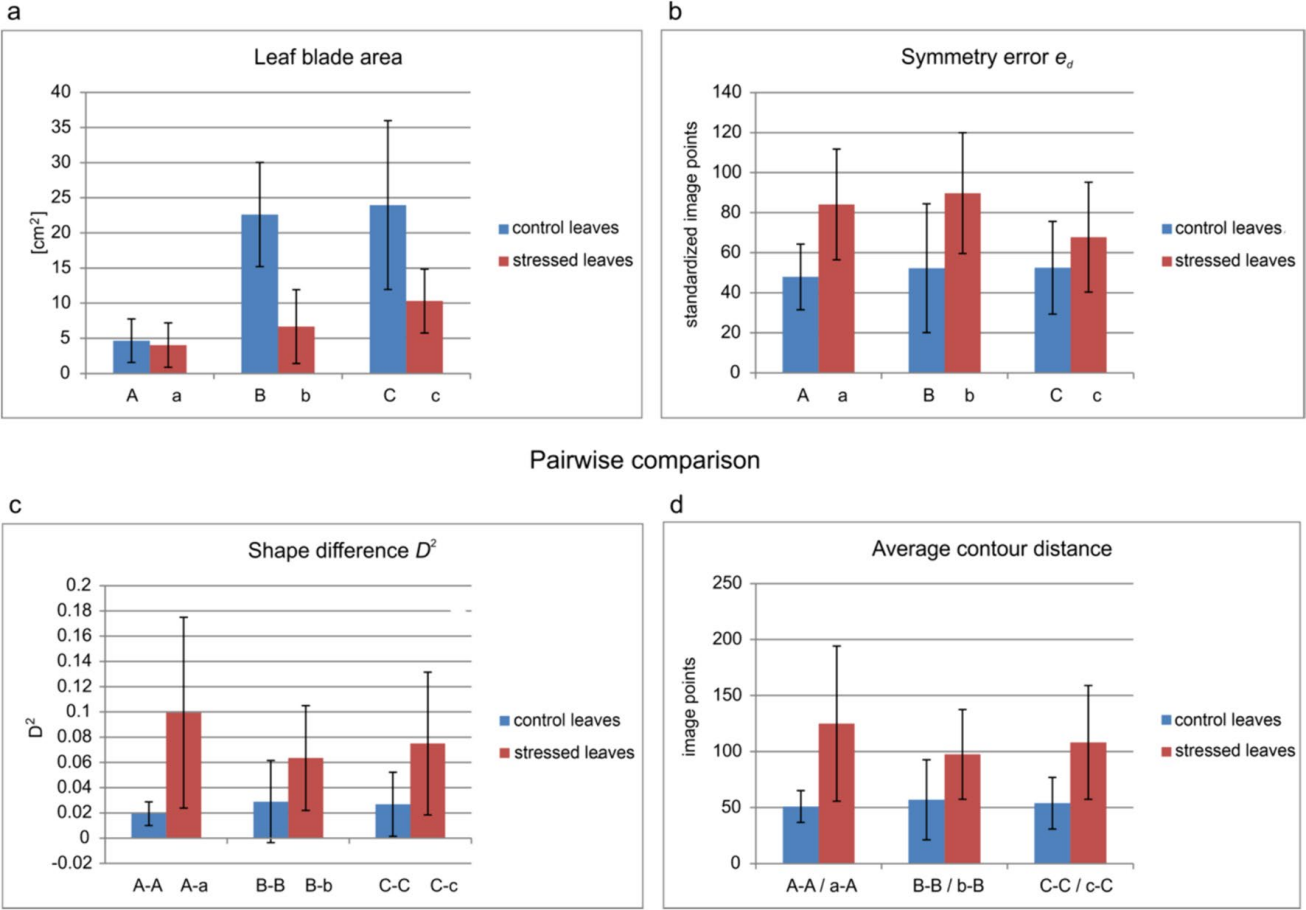
**a**–**d** The results of leaf area and symmetry analysis for control and stressed leaves. **a**, **b** Mean values ± standard error (*n* = 14). **c**, **d** Pairs of A–A, B–B, C–C: number of compared pairs *n* = 7 in each category; a–A, b–B, c–C: number of compared pairs *n* = 14 in each category

The average leaf area in the tested leaf categories is shown in Table 3 and Fig. 8a. Significant differences between the surface area of the untouched and touched leaves can be observed. Mechanical stress causes a particularly marked reduction of blade area in the nodes where the leaves are already in a more advanced growth phase. Statistical tests showed no significant differences only for pairs of leaves from the upper nodes, marked with symbols a and A (Supplementary file S2, Table S5).

**Fig. 9:**
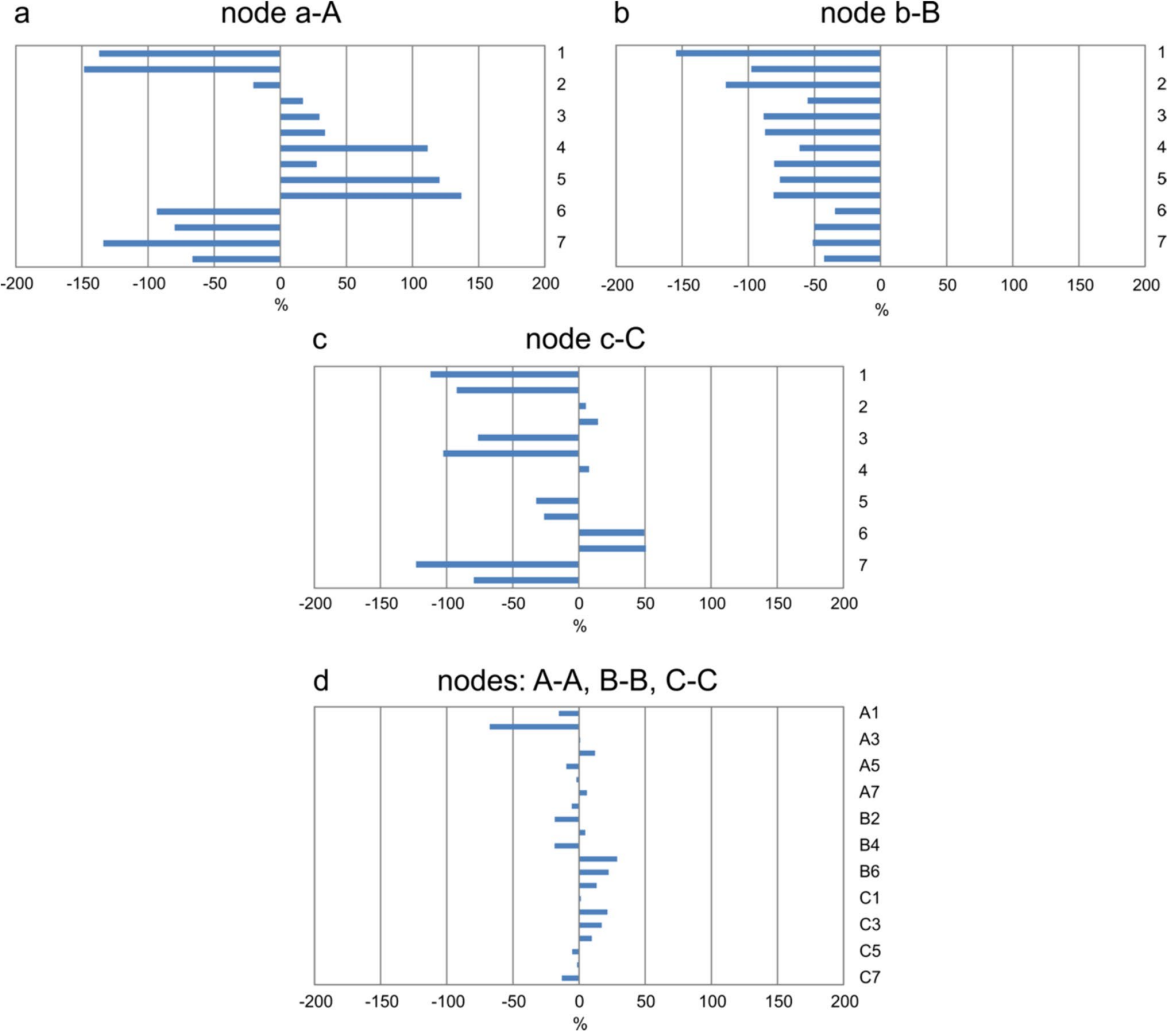
Changes in the leaf blade surface area. **a**–**c** Under mechanical stimulus in relation to the control leaves (number of compared pairs *n* = 14 in each category). **d** In pairs of control leaves coming from the same node (number of compared pairs *n* = 7 in each category)

After mechanical stimulation, leaves from the top of the plant (node a) could have both a smaller (average area loss was 4.3 cm^2^) and larger area than the control leaves (on average by more than 3 cm^2^). In the lower parts of the plant usually a smaller area of leaves was affected, compared to the control (Table 3 and Fig. 9a–c). Comparing leaves in b nodes, the average reduction in the blade area of touched plants relative to the control ones was 12.3 cm^2^. Moreover, in c nodes, reduction of the touched leaf blade was typical, with an average loss of 15.6 cm^2^. In the c–C pairs, a slight increase in the touched leaf area was observed relative to the untouched leaf, which was about 4.6 cm^2^.

For the examined pairs of leaves, the relative difference between the leaf area under the influence of mechanical stimulus and the leaf area from the control node was on average 71.6%. Differences were smaller the farther from the plant apex and amounted to 83%, 77% and 55% for pairs a–A, b–B and c–C, respectively. The relative difference in the blade area in a pair of control leaves was 14.0%. For pairs in nodes A, B and C the values were 16.2%, 15.4%, and 11.4%, respectively (Table 3 and Fig. 9a–c). Using the two-tailed Student's t-test, it was demonstrated that the relative area changes between leaf pairs of the control group ${\delta }_{1}$ and those from the combined control and touched leaves ${\delta }_{2}$ were significantly different, with a significance level of 0.05 (Supplementary file S2, Table S6). 'Pairs' refers to two leaves attached to the same node in the control group or to corresponding nodes in the control-stressed group.

### Leaf shape

As shown, nettle leaves in contact with a mechanical stimulus had an altered contour relative to untouched leaves. In this case, the average deviation of the contour reached about 100 points of the image, while in pairs of control leaves originating in the same node, these differences were about 50 points of the image. After the scale and position of the leaves were normalized, distance units have become arbitrary. Therefore, only proportions of the distance could be taken into account:

5 $${\overline{\delta }}_{avg\text{1,2}}=\frac{{\overline{d} }_{avg,1}}{{\overline{d} }_{avg,2}}=0.49,$$

6 $${\overline{\delta }}_{max\text{1,2}}=\frac{{\overline{d} }_{max,1}}{{\overline{d} }_{max,2}}=0.56,$$

where ${\overline{d} }_{avg,1}, ({\overline{d} }_{max,1})$ is the average of mean (maximum) distance of the contours in pairs of control leaves, and ${\overline{d} }_{avg,2}, ({\overline{d} }_{max,2})$ is the average of mean (maximum) distance of the contours of a control and touched leaf attached to the same node.

In addition, the maximum contour distances in the touched and control leaf pairs were almost twice as large as the maximum contour distances in the control leaf pairs. The statistical significance of the differences in the obtained results was examined without distinguishing between the nodes. The results for the examined leaf pair categories were statistically different at a significance level of 0.05 (Supplementary file S2, Table S7 and Table S8).

In the EFD frequency domain, the proportion of the mean contour distance for physical deformations (two leaves from one control pair) to the distance for pathological distortion (untouched and touched leaves) was also 0.56:

7 $${\overline{\delta }}_{EFD}=\sqrt{\frac{{\overline{D} }_{1}^{2}}{{\overline{D} }_{2}^{2}}}=0.56,$$

where ${\overline{D} }_{2}^{1}$ and ${\overline{D} }_{2}^{2}$ are the means of squared distances in the EFD transform space of contours computed for pairs of both untouched leaves and pairs of the untouched and touched leaf, respectively.

The average values of contour deformation for individual groups of nettle nodes are shown in Fig. 8c. The greatest differences in contour deviations can be observed in leaves from node a–A. The results for the examined leaf pair categories were statistically different at the 0.05 significance level (Supplementary file S2, Table S9).

### Leaf symmetry

Populations of untouched and stressed leaves were compared in terms of their symmetry, with the coefficient ${\delta }_{d}$ in Eq. (8) expressed as the average ratio of leaf contour mean distances:

8 $${\delta }_{d}=\frac{\overline{{e }_{d,2}}}{\overline{{e }_{d,1}}}=1.5816,$$

where indices 1, 2 in $\overline{{e }_{d,1}}$ and $\overline{{e }_{d,2}}$ denote averages in the compared populations of the random variables ${e}_{d,1}$ and ${e}_{d,2}$ defined in Eq. (3) (Supplementary file S2, Table S10). It was verified, using the Shapiro–Wilk test, that the random variable ${\varepsilon }_{a,1}$ of the mismatch area coefficient between untouched leaf contour and its axial symmetry mapping does not fulfill the normality condition. Therefore, the Mann–Whitney test was used to verify that the populations of ${\varepsilon }_{a,1}$ and ${\varepsilon }_{a,2}$ [Eq. (4)] were significantly different at a significance level of 0.05 (Supplementary file S2, Table S11).

Populations of touched and untouched leaves were compared in terms of their symmetry via the coefficient ${\delta }_{a}$ defined in Eq. (9), as the ratio of leaf contour mean distances.

9 $${\delta }_{a}=\frac{\overline{{\varepsilon }_{a,2}}}{\overline{{\varepsilon }_{a,1}}}=1.5841,$$

where indices 1, 2 in $\overline{{\varepsilon }_{a,1}}$ and $\overline{{\varepsilon }_{a,2}}$ denote averages in the compared populations of the random variables ${\varepsilon }_{a,1}$ and ${\varepsilon }_{a,2}$. According to the proposed coefficients ${\delta }_{d}$ and ${\delta }_{a}$, the asymmetry of leaf blades undergoing mechanical stress was assessed as 1.6 times greater than in the case of free-growing leaves. As was shown in Fig. 8b, the loss of symmetry of the touched leaves concerned each of the examined nodes.

## Discussion

It is a paradigm that environmental stress can trigger various physiological and biochemical reactions, as well as modulations of morphological processes at different levels of plant organization—from cellular and subcellular structures to the entire plant body (Lamers et al. 2020). Despite the abundance of empirical material, however, the physiological mechanism controlling these processes remains relatively poorly understood. This is mainly due to the small number of interdisciplinary experiments allowing for a comprehensive study of the effects of stress factors in highly controlled environmental conditions. We attempted this type of research, as presented in this paper.

The original experimental device used in our work was constructed specifically for our experiments and allowed us to study the impact of mechanical stress on plants under strictly controlled conditions, in terms of both the parameters of the stimulus and the surrounding environment (a growth chamber). Molecular, and anatomical techniques, as well as computer image analysis and time-lapse techniques were used to assess changes caused by mechanical impact on nettle plants. A number of reactions occurring at various levels of organization of the *U. dioica* plant body was revealed. Although direct observations of the time-lapse movies indicated that the mechanical impact of the experimental device was mainly related to the periodic touching of the leaf surface causing the stem to bend, the observed effects covered a relatively wide spectrum of reactions regarding the morphology of leaves, stem anatomical structure and gene expression in stressed plants compared to untouched control plants. It is worth noting, however, that in experiments by other authors with various plant species, in which plants organs were forced to move (e.g., by wind), they may have reacted with quite significant changes in the anatomical structure of the stem, manifested, among others, by varying thickness and height (Telewski and Jaffe 1986). In our experiment, the general anatomical stem structure of nettle plants was found to be similar in both untouched and touched plants. However, certain differences were observed in the length, shape and characteristics of the internodes. In control plants, the internodes were longer and exhibited a distinct four-ribbed structure; their shape gradually approached a rectangular form due to the secondary growth of vascular tissues.

Periodic touching of the plants with a sheet of paper did not affect epidermal pavement cells rather, the differences between the treated and non-treated plants concerned the trichomes. Control plants formed three types of trichomes that have been previously described in the literature: stinging, glandular and simple setulose trichomes (Cummings and Olsen 2011). In the stress-treated plants, stinging trichomes were not found in any of the examined sections. At this stage, we can only speculate why such adaptation to mechanical stress occurred in the present experiment. Perhaps the point is that even a light touch irreversibly destroys the stinging hairs, and thus the stressed plant is unable to use them for its proper purpose, which is to limit excessive feeding of herbivores. Supposedly, at the beginning of a multi-day experiment (when it is clear that the mechanostimulation is continuous), the touched plant sends a signal to the shoot apical meristem, i.e., to where new leaf primordia are constantly being formed, and where the pattern of all histological elements of epidermis is determined very early in the primordia. The perception of this signal results, supposedly, in the modification of gene expression so as to block the energy expenditure to create a structure that would have no chance of functioning anyway. The effect of such a mechanism would be the production of new body modules without any traces of the presence of stinging hairs.

Fluorescent microscopy observations revealed the presence of aniline blue fluorescence in setulose trichomes, indicating the accumulation of callose. Compared to control plants, Ortmannová’s ring (Kulich et al. 2015), the typical feature of trichome apoplast in some species, was more prominent in stress-treated plants, and became gradually obscured with heavy callose deposits. It is generally believed that callose positively regulates the stress response of plants (Wang et al. 2022). Callose deposition can be induced by both biotic and abiotic stresses, while deposition of this polysaccharide can also be considered as a defense mechanism in plants against pathogen attacks. Significantly, callose deposition has also been described as the reaction of a plant to mechanical stresses (Jaffe and Telewski 1984; Telewski 2006; Stass and Horst 2009).

Our research on *U. dioica* trichomes supports previous data showing that plant trichomes can sense environmental signals and respond specifically to them. Studies on *A. thaliana* have shown that trichomes can detect mechanical stimuli and convert them into physiological signals. Han et al. (2016) showed how trichomes bend under mechanical stress, while Marković et al. (2016) observed changes in trichome density in response to mechanical contact, further supporting their role as responsive elements to external signals. These results highlight the crucial role that trichomes play in plant defense and their ability to respond to various mechanical stimuli. Our research demonstrates the importance of trichomes in plant physiology and sheds light on how species such as *U. dioica* L. adapt to environmental challenges through unique mechanisms.

In our experiment, cell wall thickening and lignification were observed in pericyclic fibers in older internodes of both touched and untouched plants, but the process was initiated earlier (i.e., in younger internodes) in stress-treated plants. This result coincides with reports by some authors indicating that abiotic factors, including mechanical stresses, induce earlier lignification (Le Gall et al. 2015; Gladala-Kostarz et al. 2020) shedding new light on the relationship between mechanical stress and cell wall thickening. In the present study, no mechanical tests of the stem were performed. Therefore, we cannot claim that earlier stem lignification results in mechanical reinforcement under mechanical stress in *U. dioica*. However, we propose that the nettle lignification phenomenon might go beyond the traditional view that it is merely a mechanical process. While it provides increased stiffness to the stem and enables it to withstand mechanical stress, our results suggest that lignification may also be a general stress-response mechanism. It is known that, under certain circumstances, strategic adaptation is required that strengthens the plant's resilience to a range of stressors, including pathogenic attacks and environmental influences, among others (Bhuiyan et al. 2009). Therefore, our results are consistent with the generally accepted view that cell wall lignification is a dynamic process that is modulated at different levels during normal development and arises in response to environmental stresses, including mechanical factors (Boudet 2000).

Using image-processing methods, we demonstrated that mechanical stress significantly affects the morphology of nettle leaves, which is compatible with studies on other plant species (Jaffe 1986; Anten et al. 2010; Bano et al. 2019; Trinh et al. 2021). The average leaf area was significantly reduced in leaves subjected to mechanical stress compared to control leaves. Note that a decrease in the area subjected to a constant physical force leads to an increase in mechanical stress, as expressed by the formula σ = F/A, where σ refers to the stress, F to the applied force and A denotes the area of ​​the surface. Thus, we suggest that a change in leaf area is not an evident response to a reduction in the impact of physical force.

Our investigation of the mechanical stress response of *U. dioica* revealed leaf morphological alterations similar to those reported in *P. major* (Anten et al. 2010). In this species, mechanical stress resulted in slender petioles and elliptic and thinner laminas, whereas wind exposure in rounder and thicker leaf blades. In our study, we have demonstrated for the first time that *Urtica dioica* exhibits changes in leaf geometry when subjected to mechanical stress, which suggests an adaptation process similar to others documented in plant biology. Specifically, we observed that bilateral leaf symmetry was disrupted due to the influence of physical forces. Therefore, we suggest that it is possible to estimate the extent of physiological stress based on changes in leaf symmetry.

Leaf shape analysis revealed altered contours in stressed leaves, with larger deviations of outline and symmetry observed in touched leaves. The asymmetry of leaf blade expressed by the ratios ${\delta }_{d}$ and ${\delta }_{d}$ is about 1.5 times greater for the population of touched leaves in comparison with natural asymmetry within the control group. In our investigation of the stress response in *U. dioica*, we discovered interesting parallels with the study which examined photosynthetic efficiency in relation to leaf asymmetry in several woody species (Kozlov et al. 2019). Contrary to the traditional view that equates symmetry with optimal physiological performance, leaves exhibiting higher levels of fluctuating asymmetry may in fact contribute to more effective photosynthesis. This positive correlation between leaf asymmetry and photosynthetic efficiency, challenges the prevailing assumption that perfect symmetry is the most desirable trait for plant function. Instead, it emphasizes the adaptive flexibility of plants (e.g., *U. dioica*), which optimize leaf architecture to enhance photosynthetic performance under stress. Such adaptability may represent a broader evolutionary strategy among different plant species to maximize resource use efficiency in dynamic environments. Understanding the implications of leaf symmetry variations and their physiological effects offers valuable insights into plant resilience, adaptability, and the overall understanding of plant responses to environmental challenges.

A particularly interesting information on the reaction of nettle leaves to mechanical stress was provided by the results of the gene expression analysis. It should be emphasized that this study determined the effect of mechanical stress on the expression of only a few of the numerous genes known, in other plant species, to be involved in the regulation of the response to mechanical stress. However, even this screening of several genes revealed varied responses. Among the six studied *U. dioica* genes, *UdTCH1* showed the highest expression level, being upregulated twofold in stressed plants compared to control ones. In the same plants, *UdERF4* and *UdTCH4* were downregulated under stress conditions, with their expression levels being approximately half of those in control plants. Expression level of *UdZFP2* did not show any significant difference between stressed and control plants. Each of these responses (upregulation, downregulation, constant level of expression) may have physiological significance, because it should be assumed that the studied genes (or rather their protein products) regulate the response to mechanical stress within a network of relationships. Therefore, these results should be treated as preliminary, and research based on a full transcriptomic or proteomic profile could provide a more complete understanding. The present study suggest that *UdTCH1* may play a significant role in the nettle plant's response to mechanical stress. It remains an open question, however, whether the *UdTCH1* gene is specific to the mechanical touch response of the nettle and can be included in the group of *TOUCH* genes (Braam 2005) or whether it works more broadly in response to various types of environmental stress.

It can be concluded that the application of a mechanical stimulus to a growing nettle shoot resulted in a whole series of anatomical modifications of the stem and morphological adaptations of the leaf. This was accompanied by a significant difference in the expression level of a gene known to be involved in mechanical stress response. The results contribute to our understanding of the adaptive mechanism employed by plants to cope with mechanical stress (Kouhen et al. 2023).

We believe that studying non-model species such as *Urtica dioica* not only helps to expand understanding of plant biology, but also to adjust generalizations regarding stress responses across the plant kingdom. Therefore, we hope that our research will make a valuable contribution to existing knowledge about plant adaptability. We hope that our results inspire further research into the complex responses of plants to environmental stimuli and highlight the need to include a diverse range of species in such studies.

### Supplementary Information

Below is the link to the electronic supplementary material.

Supplementary file1 (MP4 23094 KB)

Supplementary file2 (DOCX 1459 KB)

## Data Availability

The authors confirm that the data supporting the findings of this study are available within the article and its supplementary materials.

## Abbreviations

EFD: Elliptic Fourier Descriptors
ERF4: Ethylene-Responsive Transcription Factor 4
TCH: TOUCH, touch-response mechanisms
UPS: Universal Stress Protein
ZFP2: Zinc Finger Protein 2
i: Internodes (il, the internode below the apical bud; i2, i3, i4, basipetally successive internodes)
