# Supplementary material for: The impact of mechanical stress on anatomy, morphology, and gene expression in Urtica dioica L
Source: Planta. 2024 Jul 6;260(2):46. doi: 10.1007/s00425-024-04477-0 (PMC11227470; doi:10.1007/s00425-024-04477-0)
Supplement: Supplementary file 2 — Supplementary file2 (DOCX 1459 KB) [file 425_2024_4477_MOESM2_ESM.docx]

**Supplementary file**

**Molecular analysis**

**Identification of *U. dioica* stress-related proteins**

In order to find orthologues of mechanical stress-related genes in the *U. dioica* genome, the relevant *A. thaliana* protein sequences were searched in the NCBI (NCBI Resource Coordinators 2018) and UniProt (The UniProt Consortium 2017) databases, excluding sequences described as short or redundant. Next, the orthologues were found in the *Morus notabilis* genome (the closest available sequenced relative of *U. dioica*) with the BLASTp tool (Altschul et al., 1990), using each of the *A. thaliana* protein sequences as a query. The same procedure was performed for *Prunus* sp. and *Vitis vinifera*, in order to supplement the analyses with sequences of species from sister order Vitales and from sister family Rosaceae. The sequences of putative *M. notabilis*, *Vitis vinifera* and *Prunus* sp. orthologues were used for phylogenetic analyses.

The phylogenetic analyses were performed using the Phylogeny.fr platform (Dereeper et al., 2008) and MEGA X (Kumar et al., 2018). Two methods were used: Maximum Likelihood (ML) and Maximum Parsimony (MP). The analysis performed in Phylogeny.fr consisted of the following steps:

- alignment of sequences with the MUSCLE algorithm (v3.8.31) (Edgar, 2004), with default settings and highest accuracy selected for analysis;
- phylogenetic reconstruction using the ML method in the PhyML program (v3.1 / 3.0 aLRT) (Anisimova & Gascuel, 2006; Guindon & Gascuel, 2003) or MP in the TNT program (Goloboff et al., 2008);
- graphical representation and editing of a phylogenetic tree using the TreeDyn (v198.3) program (Chevenet et al., 2006).

The analysis performed in MEGA X consisted of the following steps:

- alignment with the ClustalW algorithm (Thompson et al., 1994), with default settings and highest accuracy selected for analysis;
- phylogenetic reconstruction using a bootstrap test with a replication number of 1000 (Felsenstein et al., 1985) and the MP method using the SPR algorithm (Nei & Kumar, 2000);
- graphical representation and editing of the tree.

The trees were rooted with the relevant *A. thaliana* sequence as an outgroup.

The construction of phylogenetic trees did not bring forward any new information in the case of *USP*, *ZFP2*, *TCH1*, *TCH2* and *TCH4* (Figs S1b, c, Fig. S2). In the case of *ERF4*, the phylogenetic analysis showed that *M. notabilis* XP 024023747.1 is a closer orthologue (Fig. S1a). In the case of *TCH3*, there were two close orthologues, but *M. notabilis* XP_010087110.1 was chosen for further analysis (Fig. S2).

The *M. notabilis* genes chosen for further analyses, the primers’ sequences and RT-PCR results are presented in Supplementary Tables S1–2 and Supplementary Figure S3a. *M. notabilis* primers did not work properly; therefore, a search for *U. dioica* orthologues was necessary.

The putative orthologues in the *U. dioica* genome were searched using the BLASTp tool in the China National GeneBank DataBase. The closest orthologues were selected in accordance with the highest MAX score from the BLASTp results (Table 1). The BLASTp search demonstrated that there is one *U. dioica* orthologue for two *A. thaliana* genes, *TCH1* and *TCH3*.

**
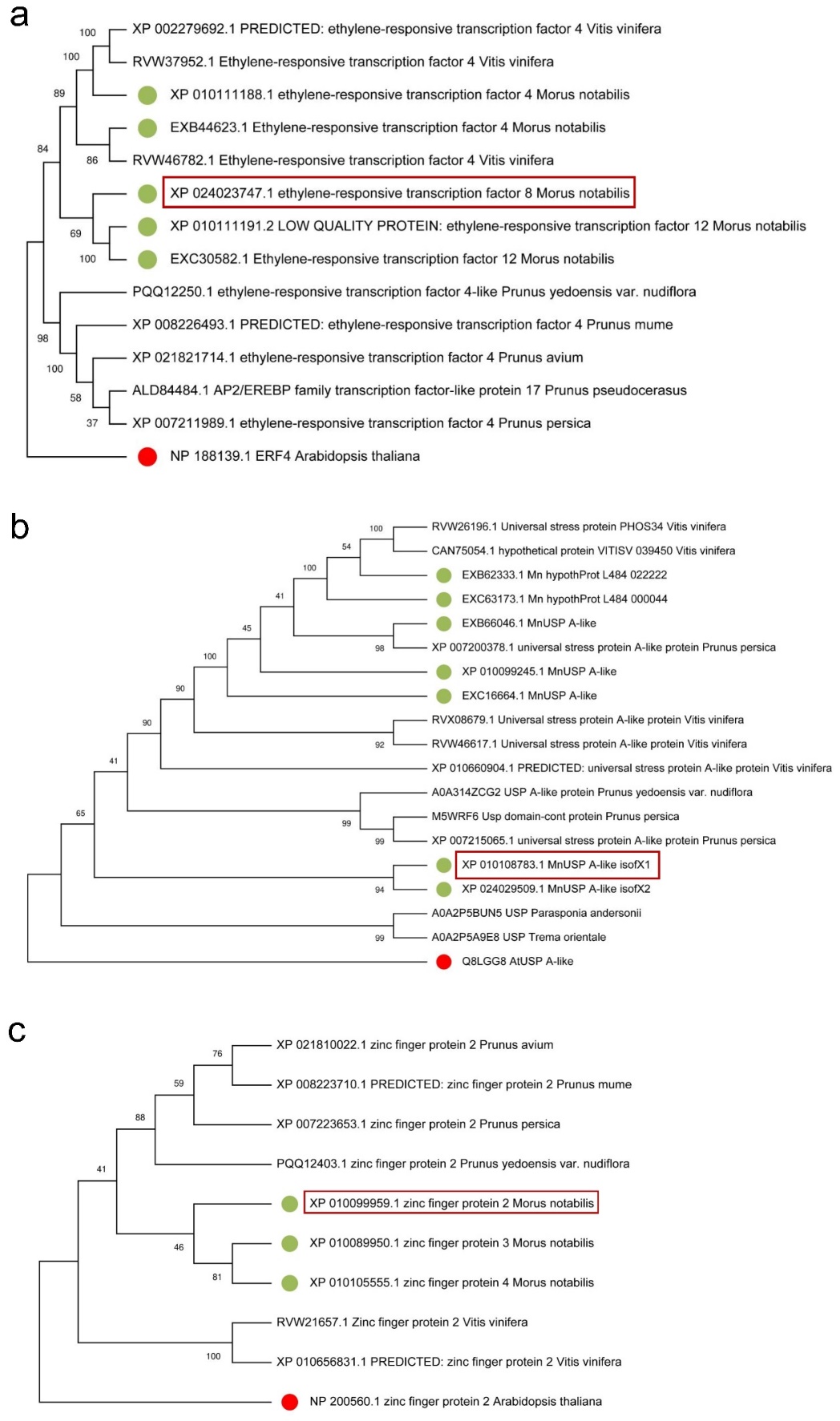
**

**Fig. S1** Phylogenetic tree of ERF4 (**a**), USP (**b**) and ZFP2 (**c**) proteins inferred using the Maximum Parsimony method. Sequences of *M. notabilis* chosen for further analysis are marked by red frames. Green dots – *M. notabilis* sequences; red dot – *A. thaliana* sequences used for rooting. The numbers next to the branches indicate the percentage of bootstrap replicated trees (i = 1000) where the associated taxa grouped together


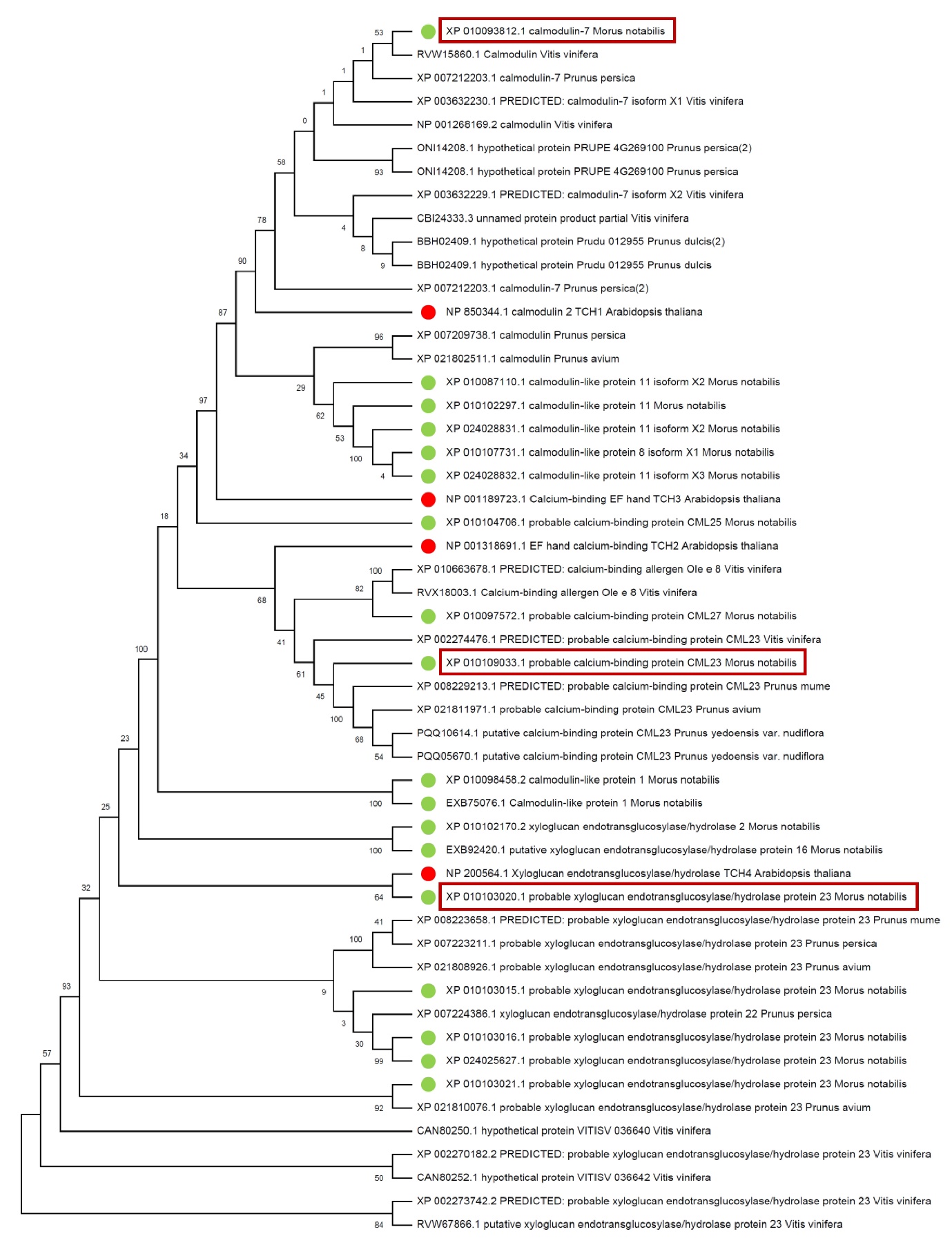


**Fig. S2** Phylogenetic tree of TCH proteins inferred using the Maximum Parsimony method. Sequence of *M. notabilis* chosen for further analysis is marked by red frame. Green dots – *M. notabilis* sequences; red dots – *A. thaliana* sequences. The numbers next to the branches indicate the percentage of bootstrap replicated trees (i = 1000) where the associated taxa grouped together

**Table S1** Sequences of mechanical stress-related proteins of *A. thaliana* and corresponding sequences of *M. notabilis* chosen for further analyses

| ***Arabidopsis thaliana*** | | ***Morus notabilis*** | |
| --- | --- | --- | --- |
| **Acronym** | **Identification number** | **Protein name in NCBI database** | **Gene name in this article** |
| ERF4 | NP_188139.1 | XP_024023747.1 ethylene-responsive transcription factor 8 | *MnERF4* |
| USP | Q8LGG8 | XP_010108783.1 universal stress protein A-like protein isoform X1 | *MnUSP* |
| ZFP2 | NP_200560.1 | XP_010099959.1 zinc finger protein 2 | *MnZFP2* |
| TCH1 | NP_850344.1 | XP_010093812.1 calmodulin-7 | *MnTCH1* |
| TCH3 | NP_001189723.1 |  |  |
| TCH2 | NP_001318691.1 | XP_010109033.1 probable calcium-binding protein CML23 | *MnTCH2* |
| TCH4 | NP_200564.1 | XP_010103020.1 probable xyloglucan endotransglucosylase/hydrolase protein 23 | *MnTCH4* |

Table S2 Sequences of primers designed for RT-PCR and Real-Time qPCR

| ***M. notabilis* gene** | **Primer** | **Sequence (5’ → 3’)** | **Product length (bp)** |
| --- | --- | --- | --- |
| *MnERF4* | Forward | GTGGTGAGTGAGCACGAGAA | 111 |
|  | Reverse | TTTACCGAAGCCACCGTCTC |  |
| *MnUSP* | Forward | TGTGCAAAGCATGCTGAGTG | 116 |
|  | Reverse | GGATGGGAAAGCCAATGCAA |  |
| *MnZFP2* | Forward | CCGTCTGAACCGACGAAAGT | 108 |
|  | Reverse | CAGCGTTCTCTCCAGCTTGT |  |
| *MnTCH1* | Forward | ACGGCACCATTGATTTCCCT | 117 |
|  | Reverse | AGCCATTCTGGTCCTTGTCG |  |
| *MnTCH2* | Forward | ACGCCTTCGATCTGTACGAC | 112 |
|  | Reverse | ATGCGAGAGCAGTCACTGAG |  |
| *MnTCH4* | Forward | TGGGCAACAAGAGGTGGTTT | 98 |
|  | Reverse | CTCCACACGCAAGAGGTCTT |  |


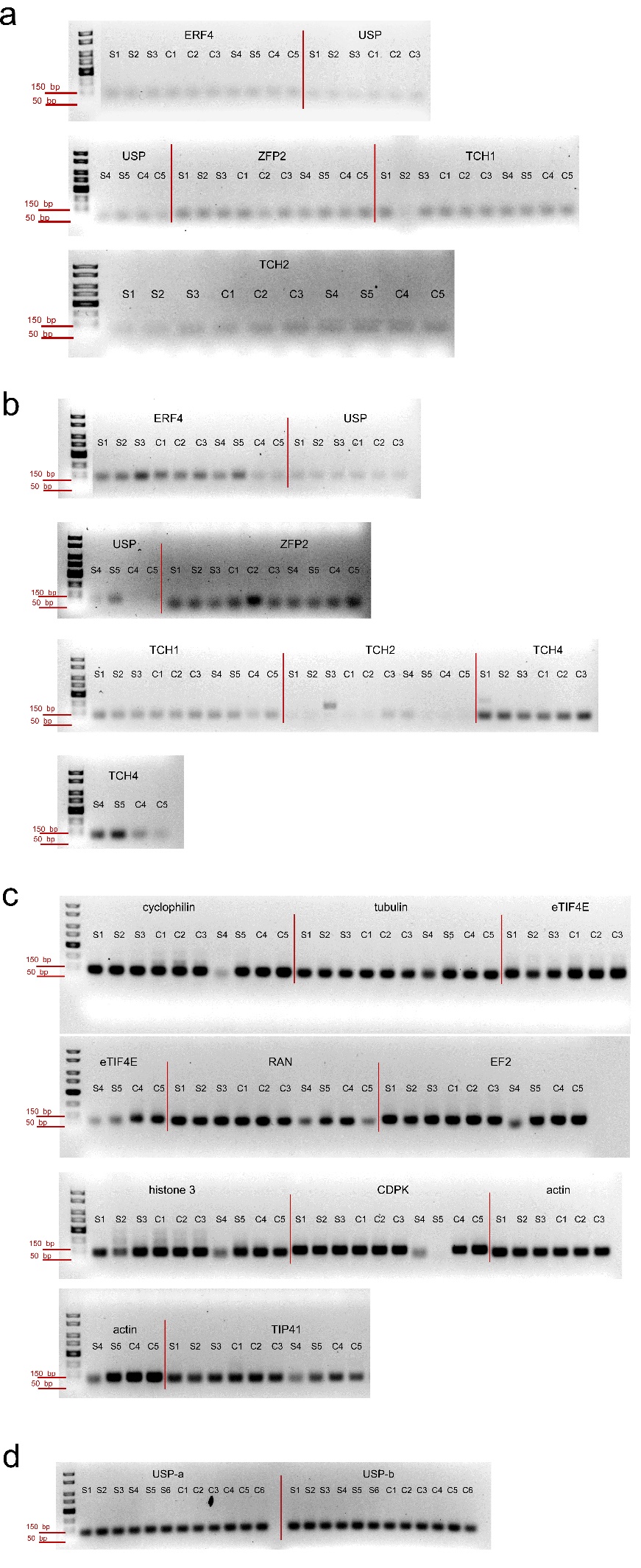


**Fig. S3** The electrophoretic separation of: (**a**) MnERF4, MnUSP, MnZFP2, MnTCH1 and MnTCH2 products of amplification performed on *U. dioica* cDNA template; (**b**) UdERF4, UdUSP, UdZFP2, UdTCH1 Ud TCH2 and UdTCH4 products of amplification performed on *U. dioica* cDNA template; (**c**) Udcyclophilin, Udtubulin, UdeTIF4E, UdRAN, UdEF2, Ud histone3, UdCDPK, Udactin and UdTIP41 products of amplification performed on *U. dioica* cDNA template; (**d**) UdUSP-a and UdUSP-b products of amplification performed on *U. dioica* cDNA template. C – control plants; S – stressed plants; bp- base pairs

**Table S3** RT-PCR conditions

| **Temperature** | **Time** |
| --- | --- |
| 95°C | 4 min |
| 40 cycles: |  |
| 95°C | 30 s |
| 60°C | 30 s |
| 72°C | 30 s |
| 72°C | 5 min |

**Statistical analysis**

**Table S4** Tests to check the statistically significant difference of the height in control and stimulated plants

| Table Analyzed | height |
| --- | --- |
|  |  |
| Column C | control |
| vs. | vs, |
| Column B | stimulated |
|  |  |
| Unpaired t test |  |
| P value | <0,0001 |
| P value summary | **** |
| Significantly different (P < 0.05)? | Yes |
| One- or two-tailed P value? | Two-tailed |
| t, df | t=13,85, df=22 |
|  |  |
| How big is the difference? |  |
| Mean of column B | 5,400 |
| Mean of column C | 17,31 |
| Difference between means (C - B) ± SEM | 11,91 ± 0,8601 |
| 95% confidence interval | 10,12 to 13,69 |
| R squared (eta squared) | 0,8970 |
|  |  |
| F test to compare variances |  |
| F, DFn, Dfd | 10,94, 11, 11 |
| P value | 0,0004 |
| P value summary | *** |
| Significantly different (P < 0.05)? | Yes |
|  |  |
| Data analyzed |  |
| Sample size, column B | 12 |
| Sample size, column C | 12 |

**Table S5** Tests to check the statistically significant difference of the leaf area [cm^2^] in tested populations of leaves (*n*=14 - sample size for each category).

| t-Test: Two-Sample Assuming Unequal Variances | |  | |
| --- | --- | --- | --- |
|  | A | a | |
| Mean | 4.67 | 4.03 | |
| Variance | 1.03E+01 | 1.08E+01 | |
| *t* Stat | 0.5265 |  | |
| *P*(T<=t) one-tail | 0.3014 |  | |
| *t* Critical one-tail | 1.7056 |  | |
| P(T<=t) two-tail | 0.6029 |  | |
| *t* Critical two-tail | 2.0555 |  | |
|  | B | b | |
| Mean | 22.61 | 6.66 | |
| Variance | 5.90E+01 | 2.22E+01 | |
| *t* Stat | 5.1053 |  | |
| *P*(T<=t) one-tail | 2.0418E-05 |  | |
| *t* Critical one-tail | 1.7171 |  | |
| *P*(T<=t) two-tail | 4.0836E-05 |  | |
| *t* Critical two-tail | 2.0739 |  | |
|  | C | c | |
| Mean | 23.96 | 10.31 | |
| Variance | 1.55E+02 | 5.01E+01 | |
| *t* Stat | 2.1949 |  | |
| *P*(T<=t) one-tail | 0.0198 |  | |
| *t* Critical one-tail | 1.7207 |  | |
| *P*(T<=t) two-tail | 0.0395 |  | |
| *t* Critical two-tail | 2.0796 |  | |
|  |  |  | |
| Shapiro-Wilk Test (α = 0.05)  H0: normal *p*-value > α |  |  | |
|  | area_1_ | area_2_ | |
| *p*-value (A, a) | 0.5868 | 0.0860 | |
| *p*-value (B, b) | 0.1252 | 0.7195 | |
| *p*-value (C, c) | 0.1095 | 0.1305 | |
|  | | |  |

**Table S6** Tests to check the statistically significant difference of the relative area difference parameter d_α_ in the tested populations: d_1_ - of control leaf pairs, d_2_ - of control and touched leaf pairs.

| t-Test: Two-Sample Assuming Unequal Variances | d_1_ | d_2_ |
| --- | --- | --- |
| Mean | 0.14036 | 0.71607 |
| Variance | 0.02116 | 0.18173 |
| Sample size | 21 | 42 |
| Hypothesized Mean Difference | 0 |  |
| df | 56 |  |
| *t* Stat | -7.8825 |  |
| *P*(T<=t) one-tail | 6.03E-11 |  |
| *t* Critical one-tail | 1.67252 |  |
| *P*(T<=t) two-tail | 1.21E-10 |  |
| *t* Critical two-tail | 2.00324 |  |
| Shapiro-Wilk Test (α = 0.05)  H0: normal *p*-value > α |  |  |
| *p*-value | 0.3178 | 0.1228 |

**Table S7** Tests to check the statistically significant difference of the parameter Mean distance of contours in pairs in tested populations of leaves. *d_avg,1_* - the population of mean distances of paired contours in control leaves, *d_avg,2_* – the population of mean distances of a control and touched leaf pairs.

|  | *d_avg,1_* | *d_avg,2_* |
| --- | --- | --- |
| Mean | 53.9315 | 110.208 |
| Variance | 607.704 | 2976.49 |
| Sample size | 21 | 42 |
| Shapiro-Wilk Test (α = 0.05)  H0: normal *p*-value > α |  |  |
| *p*-value | 0.0418 | 0.0049 |
| Mann-Whitney Test  *p*-value | 2.7466e-06 (<0.05) |  |

**Table S8** Tests to check the statistically significant difference of the parameter The maximum distance of contours in pairs in tested populations of leaves. The explanation of the parameter is included in the main text. *d_max,1_* - the population of maximal distances of paired contours in control leaves, *d_max,2_* – the population of maximal distances of a control and touched leaf pairs.

|  | *d_max,1_* | *d_max,2_* |
| --- | --- | --- |
| Mean | 177.0671 | 314.28 |
| Variance | 4933.15 | 15481.1 |
| Sample size | 21 | 42 |
| Shapiro-Wilk Test (α = 0.05)  H0: normal *p*-value > α |  |  |
| *p*-value | 0.0429 | 0.0477 |
| Mann-Whitney Test  *p*-value | 2.99923e-06 (<0.05) |  |

**Table S9** Tests to check the statistically significant difference of the parameter of the square distance between contours in pairs in the EFD transform space. $D_{1}^{2}$and $D_{2}^{2}$– the populations of squared EFD distances computed for pairs of both untouched leaves and pairs of the untouched and touched leaf, respectively.

|  | *D_1_^2^* | *D_2_^2^* |
| --- | --- | --- |
| Mean | 0.02503 | 0.07929 |
| Variance | 0.00064 | 0.0036 |
| Sample size | 21 | 42 |
| Shapiro-Wilk Test (α = 0.05)  H0: normal *p*-value > α |  |  |
| *p*-value | 6.8E-05 | 0.000386 |
| Mann-Whitney Test  *p*-value | 1.75564e-06 (<0.05) |  |

**Table S10** Tests to check the statistically significant difference of the parameter contour distance *e_d_* in two populations of leaves.

|  | *e_d,1_* | *e_d,2_* |
| --- | --- | --- |
| Mean | 50.9349 | 80.5582 |
| Variance | 25.1309 | 30.2988 |
| Sample size | 42 | 42 |
| Shapiro-Wilk Test (α = 0.05)  H0: normal *p*-value > α |  |  |
| *p*-value | 0.00016 | 0.06284 |
| Mann-Whitney Test  *p*-value | 3.36518e-06 (<0.05) |  |

**Table S11** Tests to check the statistically significant difference of the parameter area difference *ɛ_a_* in two populations of leaves.

|  | *ɛ_a,1_* | *ɛ_a,2_* |
| --- | --- | --- |
| Mean | 0.1722 | 0.2727 |
| Variance | 0.0776 | 0.1096 |
| Sample size | 42 | 42 |
| Shapiro-Wilk Test (α = 0.05)  H0: normal *p*-value > α |  |  |
| *p*-value | 0.00045 | 0.0920 |
| Mann-Whitney Test  *p*-value | 2.50056e-06 (<0.05) |  |
